# The role of the right hemisphere in semantic control: A case-series comparison of right and left hemisphere stroke

**DOI:** 10.1016/j.neuropsychologia.2016.02.030

**Published:** 2016-05

**Authors:** Hannah E. Thompson, Lauren Henshall, Elizabeth Jefferies

**Affiliations:** Department of Psychology and York Neuroimaging Centre, University of York, UK

**Keywords:** Semantic, Right hemisphere, Control, Summation

## Abstract

Semantic control processes guide conceptual retrieval so that we are able to focus on non-dominant associations and features when these are required for the task or context, yet the neural basis of semantic control is not fully understood. Neuroimaging studies have emphasised the role of left inferior frontal gyrus (IFG) in controlled retrieval, while neuropsychological investigations of semantic control deficits have almost exclusively focussed on patients with left-sided damage (e.g., patients with semantic aphasia, SA). Nevertheless, activation in fMRI during demanding semantic tasks typically extends to right IFG. To investigate the role of the right hemisphere (RH) in semantic control, we compared nine RH stroke patients with 21 left-hemisphere SA patients, 11 mild SA cases and 12 healthy, aged-matched controls on semantic and executive tasks, plus experimental tasks that manipulated semantic control in paradigms particularly sensitive to RH damage. RH patients had executive deficits to parallel SA patients but they performed well on standard semantic tests. Nevertheless, multimodal semantic control deficits were found in experimental tasks involving facial emotions and the ‘summation’ of meaning across multiple items. On these tasks, RH patients showed effects similar to those in SA cases – multimodal deficits that were sensitive to distractor strength and cues and miscues, plus increasingly poor performance in cyclical matching tasks which repeatedly probed the same set of concepts. Thus, despite striking differences in single-item comprehension, evidence presented here suggests semantic control is bilateral, and disruption of this component of semantic cognition can be seen following damage to either hemisphere.

## Highlights

• Patients with RH damage showed ‘access’ or control deficits in semantic processing.
• These deficits were in line with those previously described with LH damage, pointing to a bilateral control system.
• RH patients have difficulties summating across meanings, suggestive of subtle specialisations between the hemispheres.

## 1 Introduction

Semantic cognition allows us to understand the meaning of words and objects that we encounter and to produce appropriate thoughts and behaviour (Lambon Ralph, 2014, Patterson et al., 2007). Successful semantic cognition requires the interaction of several component processes (Jefferies, 2013, Jefferies and Lambon Ralph, 2006, Rogers et al., 2015): (i) stored knowledge about items and their features and associations (e.g., salt-pepper) and (ii) the ability to flexibly control the retrieval of information to suit our current goals and the situation. For example, when we see a sign on the motorway saying: “salt-spreading”, we are able to inhibit strong associations (e.g., dinner table) and retrieve distant associations relevant to this context (Badre et al., 2005, Noonan et al., 2010, Thompson-Schill et al., 1997). These components of semantic cognition involve distinct brain networks, and are differentially damaged in different patient populations (for example, semantic dementia vs. aphasia: Jefferies and Lambon Ralph (2006)). They may also show different degrees of lateralisation, since semantic deficits reflecting a loss of conceptual knowledge typically arise from bilateral damage, while those reflecting poor control over retrieval are largely observed following left-sided damage.

Progressive degradation of the *store* of conceptual knowledge is observed in semantic dementia, SD, following *bilateral* atrophy in the anterior temporal lobes (ATL; Bozeat et al., 2000, Galton et al., 2001, Mummery et al., 2000, Patterson et al., 2007). Several lines of evidence suggests that both ATLs support the semantic store: (i) Unlike SD patients, patients with unilateral ATL damage – e.g., through resection for temporal lobe epilepsy (Lambon Ralph et al., 2010, Lambon Ralph et al., 2012) – present only subtle semantic impairments. (ii) fMRI studies show bilateral ATL recruitment for tasks involving the comprehension of words, pictures and sounds (Rice et al., 2015, Visser et al., 2010). Nevertheless, both neuropsychological and neuroimaging studies of the contribution of ATL to picture and word semantic tasks show some degree of hemispheric specialisation, with more involvement of the left ATL for tasks involving written words and speech production (Lambon Ralph et al., 2001, Mion et al., 2010, Rice et al., 2015, Snowden et al., 2004).

The semantic deficit in semantic aphasia (SA) following *unilateral* stroke to left inferior frontal gyrus (IFG) and posterior middle temporal gyrus (pMTG; Jefferies, and Lambon Ralph, 2006) is qualitatively different to the pattern of impairment in SD. SA patients show deficits of *controlled semantic retrieval* that affect both verbal comprehension and non-verbal tasks such as picture matching and object use (Corbett et al., 2009, Corbett et al., 2009, Corbett et al., 2011, Jefferies and Lambon Ralph, 2006, Noonan et al., 2010, Thompson et al., 2015). However, unlike SD cases, SA patients show inconsistent performance across semantic tasks with different retrieval demands. They have high susceptibility to external constraints – being aided by cues and misled by miscues that direct retrieval towards or away relevant information (Jefferies et al., 2007, Noonan et al., 2010, Soni et al., 2009). SA patients also perform more poorly with strong vs. weak distracters (Noonan et al., 2010) and have difficulty selecting amongst competing items. For example, on cyclical word-picture matching tasks, performance starts well, but deteriorates when the same set of semantically-related items are repeatedly probed, such that items are targets, then become distracters, and then have to be re-selected as targets (Gardner et al., 2012, Jefferies et al., 2007, Warrington and McCarthy, 1983).

Despite semantic control being assessed across multiple neuropsychological investigations (Almaghyuli et al., 2012, Corbett et al., 2011, Jefferies and Lambon Ralph, 2006, Noonan et al., 2010, Thompson et al., 2015) and fMRI studies (Badre et al., 2005, Badre and Wagner, 2007, Noonan et al., 2013, Schnur et al., 2009, Thompson-Schill et al., 1997), the focus of the literature to date has been on the role of the left hemisphere (LH) – much less is known about the contribution of the right hemisphere (RH) to situations in which semantic retrieval must be shaped to suit the task or context. Neuroimaging studies of diverse manipulations of semantic control – including the strength of the probe-target link, the strength/number of distracters and the use of ambiguous vs. non-ambiguous words – elicit a consistent response in *both left and right IFG* suggesting that this aspect of cognition may be bilateral (Noonan et al., 2013, Rodd et al., 2005, Snyder et al., 2007, Thompson-Schill et al., 1997, Wagner et al., 2001). However, the IFG response is typically stronger and more extensive in the left than the right hemisphere (Badre et al., 2005, Noonan et al., 2013, Thompson-Schill et al., 1997). There is also an exclusively left-sided response in posterior MTG (Krieger-Redwood et al., 2015, Noonan et al., 2013), indicating that at least some aspects of controlled semantic retrieval may be left-lateralised. However, nearly all of the neuroimaging studies that contributed to the Noonan et al. meta-analysis employed words as stimuli: it is therefore unclear whether this hemispheric asymmetry reflects a bias for verbal stimuli in the left hemisphere. A recent study examined the retrieval of strong and weak semantic associations using words or pictures and found a left-sided bias for both types of stimuli (Krieger-Redwood et al., 2015). This result is consistent with the finding of severe semantic impairment for both verbal and non-verbal semantic tasks in SA patients with unilateral left hemisphere lesions.

Highly convergent evidence also points to a large-scale multi-demand executive network, which is bilateral (Duncan, 2006, Duncan, 2010, Duncan and Owen, 2000). The semantic control network overlaps with the executive control network in several key locations, including dorsolateral prefrontal cortex, intraparietal sulcus and pre-supplementary motor area (Noonan et al., 2013). This is likely to reflect the recruitment of the multi-demand network during executively-demanding semantic tasks, as well as in demanding perceptual judgements, for instance. This might explain why SA patients show high correlations between non-semantic executive tasks and high-control semantic judgements (Jefferies and Lambon Ralph, 2006). However, at least some parts of the semantic control network lie outside the multi-demand network – in particular, left anterior/inferior frontal gyrus and left posterior middle temporal gyrus show some of the strongest and most reliable responses to manipulations of semantic control and yet do *not* respond to executively-demanding tasks more generally. These components are left-lateralised (Noonan et al., 2013). Thus, demanding semantic judgements may be supported by a partially-separable bilateral executive and left-lateralised semantic control processes. Some degree of hemispheric lateralisation has also been reported within the multi-demand network: for example, right PFC has been associated with inhibition, and left PFC with selection (Aron et al., 2014, Erika-Florence et al., 2014, Hampshire et al., 2010, Mostofsky and Simmonds, 2008). Nonetheless, these constructs are difficult to separate experimentally and the current study does not set out to distinguish different aspects of executive control.

From these observations, we might anticipate that damage to right-hemisphere homologues of regions damaged in SA patients would result in some degree of semantic control deficit (since control-demanding semantic tasks activate both left and right IFG, albeit to different degrees). LH patients generally have more severe and pronounced semantic deficits than RH cases particularly for typically used semantic comprehension tasks such as synonyms tests (Robinson et al., 2010, Robinson et al., 2012). In addition to *quantitative* differences, there may also be *qualitative* differences in the contribution of the two hemispheres to semantic cognition, which could reflect differential connectivity to left vs. right ATL or language regions, or, more controversially, hemispheric differences in the nature of semantic processing (Dien, 2009, Giora, 1997, Jung-Beeman, 2005). For example, some theories suggest that the anatomy and neuronal structure of the RH is more suited to holistic, ‘summative’ processing than the LH (Chechlacz et al., 2015, Hutsler and Galuske, 2003) and that the RH activates a broader semantic field (‘coarse coding’), which can sustain the meaning of a wide range of distant associates in a coherent ‘whole’. RH patients have particular problems understanding the nuances of higher-order language, including metaphor, irony and sarcasm (Brownell et al., 1990, Foldi et al., 1983, Gagnon et al., 2003, Myers, 1983, Rehak et al., 1992, Rinaldi et al., 2004, Winner and Gardner, 1977, Zaidel et al., 2002). There is also a well-established link between impaired comprehension of emotional prosody and facial expressions and RH lesions (e.g., Blonder et al., 1991, Bowers et al., 1991, Kolb and Taylor, 2000, Kucharska-Pietura et al., 2003, Silberman and Weingartner, 1986). Some neuroimaging studies support this pattern of hemispheric difference – showing a reversal of the normal pattern of more left than right-sided activity using highly demanding verbal and picture tasks such as metaphor comprehension (Anaki et al., 1998, Brownell et al., 1990, Faust and Mashal, 2007, Giora et al., 2000, Pobric et al., 2008). Nevertheless, the results are inconsistent, with some studies arguing for an equivalent role between the hemispheres in these tasks, and others suggesting the LH still makes the most significant contribution to these tasks (Chen et al., 2008, Kacinik and Chiarello, 2007, Lee and Dapretto, 2006, Rapp et al., 2007, Schmidt et al., 2007, Yang et al., 2009).

### 1.1 This study

For the first time this study provides a comparison of semantic control deficits in stroke patients with lesions in LH and RH regions implicated in semantic control. Neuropsychological studies of impaired executive-semantic processing have almost exclusively focussed on patients with LH lesions who have damage to left inferior frontal gyrus (IFG) and/or posterior temporoparietal regions (Jefferies and Lambon Ralph, 2006, Noonan et al., 2010, Schnur et al., 2009, Thompson-Schill et al., 1998; see above). It is not yet clear whether RH patients who have damage to RIFG and temporal-parietal cortex (similar to that in SA but in the opposite hemisphere) show a loss of knowledge per se (e.g., of coarse semantic relationships), or have problems with the controlled retrieval of this information. We examined RH stroke cases on tasks that manipulated semantic control and compared their deficits with SA patients who have left hemisphere lesions, for the first time. It was predicted that: (1) RH patients would be at ceiling on basic semantic tasks, since activation in simple semantic tasks is strongly left-lateralised in frontal and temporoparietal regions (Noonan et al., 2013). However, RH patients may show impairments on tasks which have been previously associated with the RH. (2) RH patients might show executive control impairments which go beyond the semantic domain, similar to their SA counterparts. (3) On tests in which semantic deficits can be detected, RH patients might show multimodal semantic *control* deficits similar to SA cases, resulting in strong effects of cueing and distracter strength. (4) Similarly, on matching tasks designed to be sensitive to deficits in RH lesions, we anticipated declining accuracy when sets of items were presented repeatedly across several cycles such that the targets became distracters and vice versa. Patients with SA are known to show this pattern for everyday objects in both word-picture and picture-picture matching tasks (Gardner et al., 2012, Jefferies et al., 2007). Typically, repetition facilitates retrieval but this task creates a build-up of competition between semantically similar items and is therefore sensitive to difficulty guiding selection amongst competing alternatives (Gardner et al., 2012, Schnur et al., 2006).

## 2 Methods

### 2.1 RH patients

There were 9 RH stroke patients, recruited from stroke clubs in Yorkshire, UK. Patients were selected for the study if they showed impairments on two or more subsets of the Right Hemisphere Language Battery (Bryan, 1995). They were not specifically selected on the basis that they showed multimodal deficits or deficient semantic control. Patients were also excluded if they showed signs of visual neglect (see below). All cases had a CVA at least one year prior to testing. Demographics for RH patients are displayed in Table 1. There were no differences between the RH and LH patients (SA and mild SA cases) on any of these characteristics (t<1, n.s.).

### 2.2 SA patients

The RH patients were compared where possible with SA and mild SA patients who have LH damage. We compared performance on background tasks to a cohort of 21 SA cases, many of whom have been previously described (Jefferies and Lambon Ralph, 2006, Thompson et al., 2015). They were recruited from stroke clubs and speech and language therapy services in Manchester and York, UK. SA patients were selected to show deficits in both word and picture association tasks, and had chronic impairments resulting from a CVA at least one year prior to testing. Not every case completed every task; details are provided in Table 3, Table 4. These patients were not tested on the tasks we specifically designed for the RH group (2, 3 of this paper) because pilot testing suggested they showed floor-level performance on these challenging semantic assessments.

### 2.3 Mild SA cases

Since the RH patients performed well on standard semantic assessments, we also had a comparison group of 11 high-functioning ‘mild’ SA cases. Three of these cases were described previously (Hoffman et al., 2011). Lesions for the mild SA cases are presented in Table 2. All 11 mild SA cases had some degree of aphasia, but they were at normal or borderline levels on background semantic tasks. This group nevertheless showed some degree of impairment on semantic tasks with pronounced control deficits on tasks taken from Noonan et al. (2010) – and they showed effects of cueing, semantic association strength and an inability to ignore irrelevant associations.

### 2.4 Controls

There were 12 healthy control participants, age- and education-matched with the patients. Participants had no history of brain injury, and showed unimpaired cognitive functioning on the Mini-Mental State Examination (Folstein et al., 1975).

### 2.5 Stroke lesion analyses

Both LH and RH stroke rarely produce lesions of inferior parts of ATL, since this region of cortex receives a dual blood supply from the MCA and anterior branch of the PCA (Phan et al., 2005, Phan et al., 2007). Stroke is even less likely to produce bilateral damage in this region (Borden, 2006, Conn, 2003). The RH patients were therefore expected to have damage focussed on frontal or temporal-parietal areas similar to SA cases but in the opposite hemisphere.

**Fig. 1:**
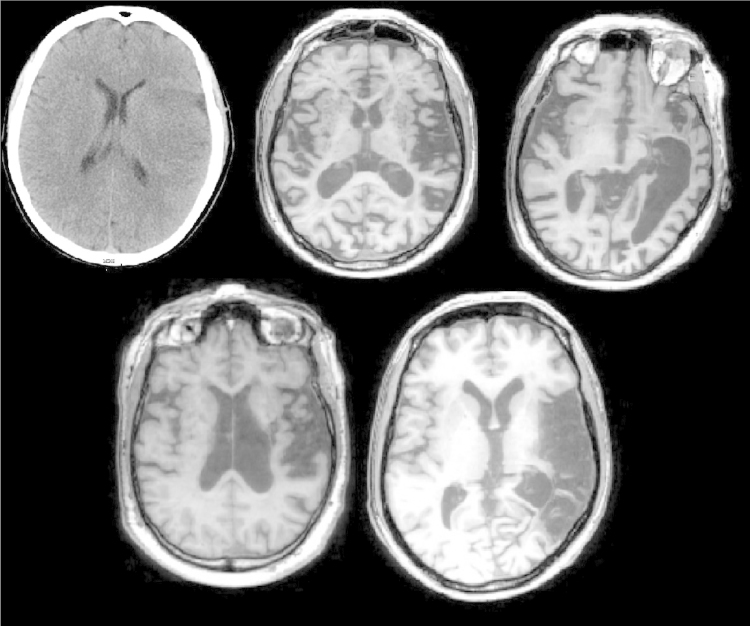
Individual RH lesions CT/MRI scans, showing the slice with the greatest lesion. From top left: ASW, DGX, SYN, NDW, DNQ.

CT/MRI scans that were available are shown in Fig. 1. These were manually traced onto Damasio’s standardised templates (Damasio and Damasio, 1989). In Table 2, to help comparisons between patient groups, we show lesion data from RH cases and mild SA cases, plus summary information for 15 SA cases where scans were available (since most of these lesions were described previously). Some of the RH and mild SA patients were unable to be scanned, but provided a radiographers report. Two mild SA cases had lesions described in Hoffman et al. (2011). JB had damage to temporoparietal cortex, and ABU had an enlarged ventricle and reduced grey-white matter contrast in the basal ganglia. JHU and ONY were not scanned. In our RH group, there were three radiographers' reports (ESP was never scanned). Reports revealed the following: RHE had a large, established infarct in the territory of the right middle cerebral artery, and an occlusion of the right inferior cerebral artery. DSW had an infarct of right temporoparietal cortex, and effacement of sulci. BWJ had thrombus to the right internal carotid artery, leading to damage of deep ganglionic nuclei, internal capsule and anterior temporal lobe, and right hemispheric sulci. While this description of BWJ's brain damage suggests that he might not have had damage to regions strongly implicated in semantic control, inspection of the data revealed that BWJ's semantic deficits were no different from those of the other RH patients. Excluding him from the sample made little difference to the outcome of analyses. In summary, SA, mild SA and RH showed similar damage (albeit in the left and right hemispheres respectively). There were no significant differences in the degree of damage in any Brodmann areas between any group (analyses shown in Supplementary materials). All groups had damage focused on lateral prefrontal and posterior temporal areas.

## 3 Background neuropsychology

### 3.1 Right Hemisphere Language tasks

*The Right Hemisphere Language Battery* (RHLB, Bryan, 1995) was used to select cases suitable for study (e.g., those RH patients with a deficit in two or more subtests). There were six subtests, described below. In all cases where reading was involved, the experimenter read aloud the written sentences.

(i) Word and picture metaphor tasks. The word task involved matching a probe sentence with one of three spoken sentences for its correct interpretation. For example: *“Probe: He didn’t take the changes lying down. Options: 1. He didn’t want to lie down. 2. He protested against the changes. 3. Change made him tired”.* In the picture condition, there was a spoken sentence to be matched with one of four pictures, one distractor being a literal interpretation of the sentence. There were ten items in each test.
(ii) An inference test: answering questions about a short story which required inference from the text. This used three paragraphs (and a practice paragraph) with four questions about each paragraph.
(iii) 20-item word-picture matching, presented with five distractor items (some of which were semantically, phonologically or visually related to the target). RH cases were not expected to be impaired on this simple task.
(iv) A 10-item humour test: selecting which of four possible sentences was the best punch line for the joke. For example: “*A judge had just finished telling the prisoner that he was free to go, as the jury found him not guilty of fraud. The prisoner then asked…(A) When can I leave sir? (B) What about my friends? (C) Does that mean I can keep the money? (D) What time is it please?*”
(v) A 10-item test of emphatic stress, where the researcher read a sentence which described a picture and the participant then described a similar picture using the same prosody.

*The Familiar and Novel Language Comprehension test* (FANL-C, Kempler and Van Lancker, 1985), used 20 spoken sentences in each condition, with a four-choice picture test of (i) novel literal phrases and (ii) familiar metaphoric phrases. A sentence was presented verbally and participants were asked to pick which picture out of four options reflects the sentence (e.g., metaphoric – ‘*he’s got his head in the clouds’*; literal – ‘*he’s chasing after a white duck*’).

### 3.2 Semantic tasks

We ran a standard battery of semantic tasks sensitive to deficits in SA cases.

(i) *The Camel and Cactus task,* picture version (CCTp; Bozeat et al., 2000) used 64 items, and involved matching a probe picture with an associated picture, presented alongside three distracter pictures (e.g., does camel go with cactus, tree, sunflower, or rose?).
(ii) Category and Letter fluency: patients were given a minute to name as many ‘*animals’* as they could (category fluency), and items beginning with ‘*S’* (letter fluency).
(iii) *A synonym judgement task* involved matching a probe word with a target word presented alongside two unrelated distractors. This had 96 items in two frequency bands (high and low) and three imageability bands (high, medium and low), producing sixteen trials in each of the six frequency-by-imageability conditions (see Jefferies et al. (2009)). All of the words in each trial were in the same frequency/imageability bands. For example, a low imageability, low frequency item involved matching suffix with inflection, perpetrator or temerity, while a high imageability, high frequency item involved matching money with cash, car or church. Responses were untimed.

### 3.3 Visual tasks

Visual neglect and visual processing were assessed in RH patients using the following tests.

(i) *The Visual Object and Space Processing battery, VOSP* (Warrington, and James, 1991): All eight subtests and the screening test of these perceptual tasks were presented. Four measure visual object perception (Incomplete Letters, Silhouettes, Object Decision, and Progressive Silhouettes) while the other four measure visual space perception (Dot Counting, Position Discrimination, Number Location, and Cube Analysis).
(ii) *The Bells Cancellation test* was used to assess visual neglect. Patients attempted to find 35 images of a bell amongst distractors distributed across a sheet of paper (Gauthier et al., 1989). Patients were not limited in time to complete this test (although RT was recorded). This was scored by counting how many bells were marked on each side of the page.

### 3.4 Executive tasks

The same executive tasks were presented to RH and SA patients.

(i) *Forward and backward digit span* (Wechsler, 1987), assessed auditory working memory.
(ii) *Elevator Counting* involved counting tones in two conditions. In the ‘no distraction’ condition, all of the tones were targets to be counted. In the ‘with distraction’ condition, patients were asked to count low pitch tones and ignore high pitch tones. This test was taken from the Test of Everyday Attention (TEA; Robertson et al., 1994)
(iii) *The Hayling Test* involved completing spoken sentences with single words (Burgess, and Shallice, 1997). Participants were asked to complete the sentence with either a sensible word or an unconnected word, in two conditions, e.g., *“She posted the letter without a…” “stamp/tomato”*.
(iv) *Ravens Coloured Progressive Matrices test* (RCPM: Raven, 1962), assessed non-verbal reasoning using pattern and rule recognition with shapes and colours in sets A, AB, and B.
(v) *The Brixton Spatial Rule Attainment task* (BSRA: Burgess, and Shallice, 1997), involved making predictions about the movement of a dot, based on patterns that it showed across trials, and then adapting these predictions when the pattern changed.
(vi) *The Trails Test* required participants to draw a line between letters and numbers in order, in an easy condition (part A, e.g., 1-2-3…) and difficult condition (part B, e.g., 1-A-2-B-3-C…, Reitan, 1958).

## 4 Results

Scores on the background language and semantic tests are shown in Table 3. Where possible, SA and mild SA patients also completed the tasks (although SA patients with particularly poor semantic comprehension were not able to complete some of the more advanced tests, such as metaphor comprehension).

### 4.1 Right hemisphere language

In a comparison of word and picture metaphor comprehension in RH and mild SA patients, using tasks from the Right Hemisphere Language Battery (RHLB, Bryan, 1995), there was a main effect of modality: F(1,14)=27.879, p<.001, and an interaction of modality and group: F(1,14)=8.605, p=.011, but no main effect of group. Mild SA patients performed similarly on word and picture metaphor tasks, while RH patients were disproportionally impaired at the picture metaphor task, in line with previously findings (Winner and Gardner, 1977). In all other subtests of the RHLB, there were no noticeable differences between groups (both RH and mild SA patients showed some impairment).

In a similar comparison of literal and metaphorical picture matching (the FANL task), there was a main effect of sentence type: F(1,13)=10.024, p=.007, an interaction of sentence and group: F(1,13)=7.459, p=.017, and no group difference: F(1,13)=2.321, p=.152. Mild SA patients performed similarly in metaphorical and literal sentences, while RH patients were poorer at metaphor sentence comprehension.

### 4.2 Semantic tasks

RH patients performed at a normal level on basic verbal and pictorial semantic tasks, and on assessments requiring both comprehension and speech production. In a comparison of SA and RH patients, performance was significantly higher in RH patients in all semantic tasks: CCTp: t(26)=4.088, p<.001, synonym judgement: t(27)=4.618, p<.001, and category and letter fluency: t(25)≥6.770, p<.001. Even mild SA patients were significantly worse than RH patients on the synonym judgment task: t(14)=3.510, p=.003, and category and letter fluency: t(14)≥2.962, p≤.010. In contrast, there were no significant differences between RH patients and healthy controls on any semantic task.

### 4.3 Visual tasks

Visual and executive task performance is shown in Table 4. RH patients were within the normal range on the majority of subtests of the VOSP. There was no difference between RH and SA or mild SA patients on this battery of visual tests. RH patients also showed no evidence of visual neglect, as measured by the Bell cancellation task (Gauthier et al., 1989).

### 4.4 Executive tasks

All RH patients individually showed abnormal performance on some executive tasks. There was no difference between RH and SA patients on the majority of tasks: t(17)≤1.903, p≥.074 for the following: Trails B (difficult condition); Brixton Spatial Rule Attainment (BSRA); Raven’s Coloured Progressive Matrices (RCPM) and Elevator Counting with distraction. Where differences approached significance, RH patients scored more highly than SA cases. At the RCPM, RH cases were worse at set B than set A (t(8)=4.000, p=.004) and set AB (t(8)=3.780, p=.005), reflecting the progressive difficulty of this task. There was also a difference in some ‘easy’ versions of executive tasks (Trails A and Elevator Counting without distraction), which likely reflected poorer basic language skills in the SA cases: t(17)≥2.777, p≤.013. The SA patients had lower forward and backward digit span (t(26)≥2.106, p≤.045), as has been found previously (Laures-Gore et al., 2011). Interestingly, although both groups were impaired at the Hayling sentence completion task for unconnected endings, the RH cases performed more poorly than the SA cases: t(18)=4.428, p<.001. For sensible ending sentence completion, the RH cases performed better: t(19)=2.702, p=.014, which may reflect omission errors in the SA group due to language problems. Therefore, there was a significant group by condition interaction on this test: F(1,18)=44.600, p<.001.

A comparison of RH to mild SA cases revealed similar findings: no difference on Trails A or B, BSRA, RCPM, digit span (forwards or backwards), or TEA with distraction: t(15)≤1.348. There was a significant difference on the easy version of the TEA: t(12)=3.177, p=.008, reflecting better performance in RH patients.

### 4.5 Summary

RH patients showed problems with metaphors, particularly when they were depicted as pictures, supporting previous findings (Winner, and Gardner, 1977). In all semantic tasks involving basic comprehension of single items, however, they showed ceiling-level performance. In terms of executive control, the three groups of RH, mild SA and SA patients were similarly impaired. Any significant differences largely reflected SA patients' greater language deficits. However, SA patients were better than RH cases at completing sentences with unconnected words. This might reflect a particular difficulty with using a strategy to overcome a deficit of verbal suppression in RH cases (Robinson et al., 2015).

## 5 Complex semantic tasks

### 5.1 Rationale

In the previous section, RH patients showed better performance than SA patients on standard semantic tasks, suggesting a *quantitative* difference between the groups. In a few tasks, however, RH cases showed a greater impairment than expected – e.g., in picture metaphor comprehension and the inhibition of prepotent sentence endings – consistent with a *qualitative* difference between SA and RH cases. To test this possibility, we examined a challenging semantic ‘buffer’ task, which mild SA cases with subtle impairment of semantic control have been shown to fail previously (Hoffman et al., 2009). We compared our RH sample with this previously-collected data from three mild SA, six SA cases and ten controls. The task involved listening to a string of words, followed by a probe word, and responding ‘yes’ or ‘no’ to indicate whether the probe word was in the same category as *any* of the previous words (e.g., *cup, cat, daffodil*; probe: *dog* → ‘yes’, as both cat and dog are animals) (Martin et al., 1994). Mild SA cases performed poorly on the semantic version of this task, despite showing normal performance on a non-semantic version involving rhyme judgements (e.g., *dog, wish, hat*; probe: *dish* → ‘yes’) (Hoffman et al., 2009, Hoffman et al., 2011). The sensitivity of this task to mild deficits of semantic control is thought to reflect the need to respond on the basis of categorical overlap (whilst avoiding potential matches based on personal or semantic associations) for multiple items in memory (Hoffman et al., 2012). If the distinction between the impairment in SA and RH cases is one of degree, RH cases should show disproportionate difficulty on the semantic compared to the phonological task, like those with mild SA (even though their performance might be at a higher level overall). Alternatively, if RH patients have semantic deficits that are focussed on particular modalities or domains of conceptual processing, they might not show impairment even on this very challenging task, since it involves judgements about a series of individual words and there is no need to identify a holistic meaning based on weak relationships between all of the words (i.e., no requirement to ‘summate’).

### 5.2 Method

Materials were taken from Hoffman et al. (2009) and based on those used by Martin et al. (1994). The semantic category task involved 189 items in nine categories: animals, body parts, clothing, flowers, fruits, insects, kitchen items, trees and types of weather. The non-semantic condition involved making rhyme judgements about words. There were word lists of increasing length, up to nine words. Each list was read aloud, followed by the probe word. Participants then responded ‘yes’ or ‘no’ to indicate whether the probe was in the same category/rhymed with any of the preceding words. List lengths of 1, 2 and 5 had 20 trials while the remainder had 24 trials. Patients had to be at least 75% accurate on each list length in order to proceed to the next list length. Span was defined as the maximum length at which the accuracy was 75% or higher, and if this came between two lengths, span was calculated with linear interpolation. Prior to beginning the semantic buffer task, participants were given a list of the categories and were asked to assign each item to one of the categories. If they made an error, the experimenter told them the correct category. The categorisation accuracy of the RH patients was 98%; mild SA scored 94%.

### 5.3 Results

**Fig. 2:**
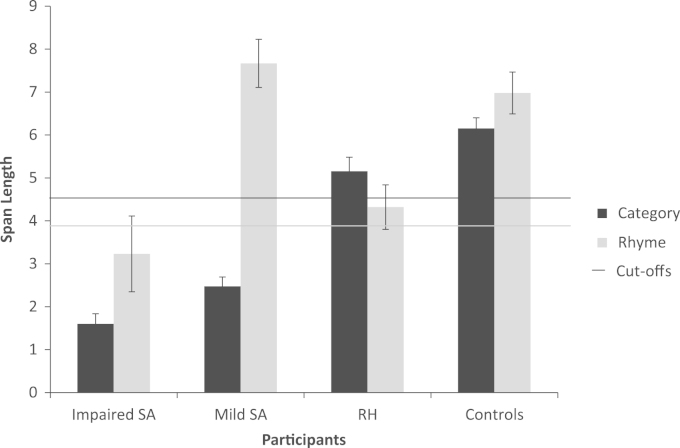
Performance on the semantic and rhyme buffer tasks. Error bars show standard error of mean.

Performance on these tasks is given in Fig. 2. Data from healthy controls, SA and mild SA patients is reproduced from Hoffman et al. (2011). In a comparison of RH and mild SA cases, there was no main effect of group (F<1). There was a main effect of task (semantic vs. rhyme): F(1,10)=21.598, p=.001, and an interaction: F(1,10)=40.401, p<.001. Mild SA patients were strikingly better at the rhyme version of the task, and poor at the semantic version, while RH patients showed similar performance in both tasks. In a comparison of RH and SA patients, there was a main effect of group: F(1,13)=21.221, p<.001: SA patients showed floor effects. There was no main effect of task (F=1.1), but again there was an interaction: F(1,13)=10.141, p=.007. SA patients resembled mild SA cases, albeit with a lower overall level of performance.

Additionally, we also looked at performance in each RH patient using the revised standardised difference test (RSDT; Crawford et al., 2010), which examines the difference between two tasks (rhyme vs. semantic) for a patient, in relation to controls (using a modified t-statistic). None of the RH patients showed a difference between the semantic and rhyme conditions that varied from controls (p>.05). This was in contrast to the mild SA cases, who all showed larger difference between the semantic and rhyme conditions than controls (p<.01). Despite floor effects, all but one SA patient (NY) also showed this difference (p<.05).

### 5.4 Summary

Even on a demanding semantic task, which required retrieval of categories that were not explicitly revealed for multiple items held in memory, RH patients showed performance in the normal range. This supports the suggestion that there may be some distinction in the contribution of the two hemispheres to semantic processing. Our RH patients showed no impairment of single word comprehension (see also Klepousniotou and Baum (2005)) and the semantic buffer task similarly required words to be categorised individually, even though several items were presented in each trial (i.e., the meaning of one word did not influence the categorisation of the next item).

In the remainder of the empirical work, we focus on conceptual domains that have revealed greater impairment in RH cases to establish whether there are any parallels with the deficit of semantic control in SA. Patients with SA retain conceptual knowledge that they nevertheless fail to retrieve in tasks with high control demands, when there are few external cues to constrain retrieval. They show strong cueing and miscueing effects (Jefferies et al., 2008, Noonan et al., 2010, Soni et al., 2009, Soni et al., 2011). Additionally, they show poorer retrieval with strong distractors which compete with the target (Noonan et al., 2010). In the next experiment, we examined the effect of cueing, miscueing and distractor strength on performance in RH cases using paradigms designed to be sensitive to deficits in this group. Since previous studies have suggested that RH cases have difficulty ‘summating’ meaning across multiple inputs, and with interpreting emotional faces (Adolphs, 2002, Nakamura et al., 1999, Witteman et al., 2011), we manipulated control demands in these tasks.

## 6 Summation and task-irrelevant information

Beeman et al. (1994) and Jung-Beeman (2005) has suggested that the RH shows ‘coarse semantic coding’ grouping together disparate words into an overarching meaning (e.g., eyes – closed – night → sleep; or foot – cry – glass → cut). Visual field experiments have revealed faster processing of distantly related words in the ‘RH’ (or left visual field) comparison to the ‘LH’ (Anaki et al., 1998, Faust and Mashal, 2007, Mashal and Faust, 2009), and patient studies have revealed difficulties with semantic comprehension of distant relationships after RH damage (Blake et al., 2015, Tompkins et al., 2011, Tompkins et al., 2008). We varied the semantic control demands within a 4 alternative forced choice (4AFC) ‘summation’ task by (i) providing cues and miscues that either led retrieval towards or away from relevant aspects of knowledge, and (ii) by manipulating the identity of the distracters. If RH cases show strong sensitivity to distractor type and cueing like SA cases, this would suggest that they retain semantic knowledge that they cannot always retrieve appropriately. We also compared comprehension within this task using words and pictures to investigate if RH cases have a multimodal deficit of semantic control, like those with SA.

### 6.1 Rationale and procedure: cues and miscues

**Fig. 3:**
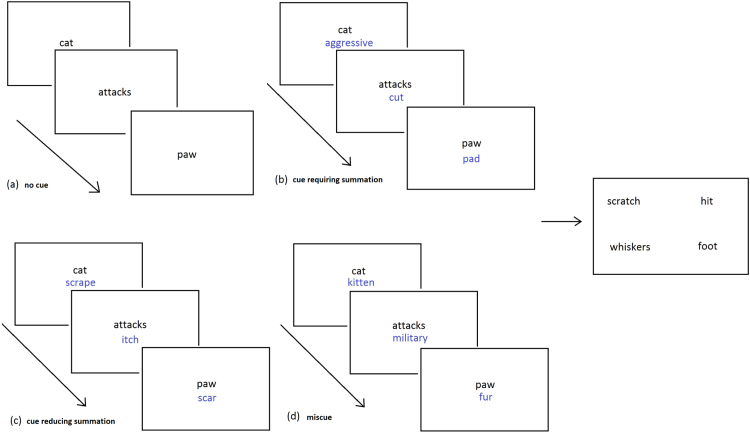
Example trials from the summation task paradigm: (a) no cue, (b) cue requiring summation, (c) cue reducing summation, (d) miscue. The target (scratch) and distractors are presented in the right panel.

We used stimuli from Beeman et al. (1994). Participants were asked to pick a target word amongst distractors. The probes were three weak associates of the target (e.g., cat-attacks-paw → scratch). The probe words were presented one at a time (for 1 s each), accompanied by cue words in cueing conditions, and then the target with 3 distractors appeared (see Fig. 3). These were presented for an unlimited time until a response was made. Distractors were related to the probes (e.g., hit, whiskers, foot, scratch). There were four conditions: (i) no cues, (ii) cues requiring summation, (iii) cues reducing summation, and (iv) miscues. All 4 conditions had the same probes, target word and distractors. (1) The first type of cue was designed to strengthen the retrieval of relevant aspects of meaning for *individual* probe words but *not* to reduce the requirement to summate across the probes (indeed, the summation requirement may have been increased since patients were required to combine the cue words and the probes). For example, the cue *aggressive* was used to link the probe word cat with target scratch, in the trial cat *aggressive*, attacks *cut*, paw *pad* → scratch, hit, whiskers, foot. (2) The second type of cue reduced the summation demands by more directly priming the target word but not the appropriate meaning of the probe words, as in the trial cat *scrape*, attacks *itch,* cut *scar* → scratch. Cues in the ‘reducing summation’ condition were significantly more associated to the target than the cues in the ‘requiring summation’ condition (t(93)=2.922, p=.004), using the Edinburgh Associative Thesaurus (Kiss et al., 1973) and the University of South Florida word association norms (Nelson et al., 1998) where available. (3) We also presented miscues which directed attention *away* from relevant aspects of each probe concept, e.g., cat *kitten*, attacks *military*, paw *fur* → scratch.

Cues are thought to benefit SA patients because they reduce the need for internally-generated constraint over semantic retrieval, allowing information to become accessible (Corbett et al., 2008, Jefferies et al., 2008). SA patients are also highly sensitive to miscues that activate irrelevant information and increase the need to select/inhibit semantic features (Noonan et al., 2010, Soni et al., 2009, Soni et al., 2011). Thus, SA patients should have higher accuracy in the two cue conditions and poorer performance following miscues. If RH patients have a similar deficit in controlling the spread of activity within the semantic system (yet a relatively intact store of conceptual information), they should also show poorer performance following miscues than cues – while controls should show good performance across conditions.

However, in addition to this predicted similarity between SA and RH cases (emerging from the hypothesis that both hemispheres contribute to semantic control), subtle differences might be expected across the two cueing conditions. The ‘cues requiring summation’ condition facilitates selection of the relevant features of each probe word but introduces additional words that are not strongly related to the target, potentially increasing the need to inhibit irrelevant information. SA cases might benefit from both types of cues (since cues in both conditions reduce the need to generate internal constraints that can shape retrieval to suit the task), while RH cases might not benefit from cues requiring summation (reflecting difficulties with inhibition/summation).

### 6.2 Results: cues and miscues

**Fig. 4:**
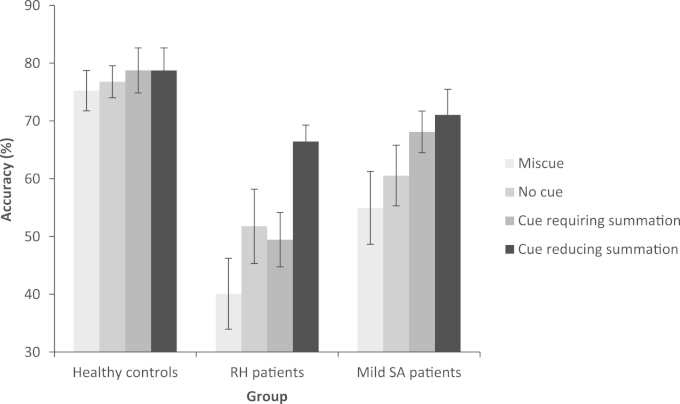
Effect of cue condition in the summation task in controls, RH patients and mild SA patients. Error bars show standard error of the mean.

Results were available for 9 RH, 8 mild SA cases and 12 controls (Fig. 4). Controls were not tested on the easiest ‘cue reducing summation’ condition. An ANOVA comparing RH cases to controls for three cue conditions (cue requiring summation, miscue, or no cue), found there was a significant effect of cue: F(2,18)=6.224, p=.009, group: F(1,19)=27.533, p<.001, and an interaction of cue type and group which approached significance: F(2,18)=3.076, p=.071. This reflected marginally greater effect of miscues in RH cases than healthy controls. RH patients were impaired at all versions of the task in relation to controls (t≥3.914, p≤.001).

A comparison between patient groups (RH patients and mild SA), examining four cue conditions (cue requiring summation, cue reducing summation, miscue or no cue), showed a significant effect of cue type: F(3, 12)=8.301, p=.003, an effect of group that approached significance: F(1,14)=3.422, p=.086 (SA>RH), and no interaction of group and cue type: F(3,12)=1.436, p=.281. Overall, the groups responded to cueing in largely the same way. There was no difference (interaction) between the groups when comparing cues reducing summation with the miscue or no cue conditions (F<1.4, p≥.257). The nearest group difference was comparing cues requiring summation and cues reducing summation, the interaction between group and cue type approached significance: F(1,14)=3.832, p=.071. This reflects a marginally bigger difference in the cue type for RH patients than mild SA cases (RH patients appeared to benefit marginally more when the cues did not require summation). Nonetheless, the patients are largely indistinguishable in their performance on this task.

### 6.3 Rationale: modality

**Fig. 5:**
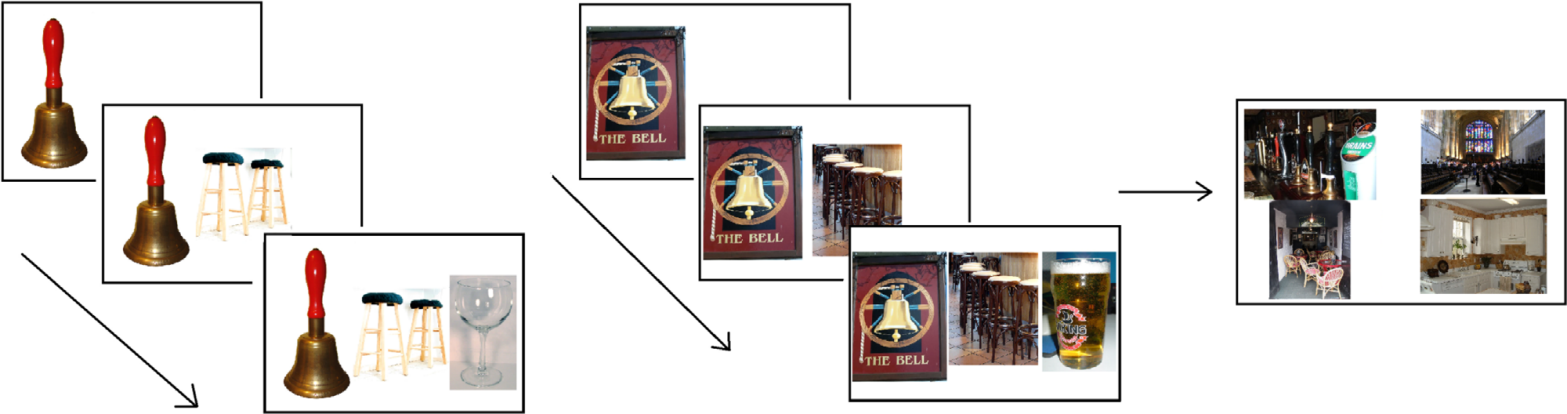
Example of a picture summation condition. On the left is the ‘no context’ condition, in the middle the ‘with context’ condition, and the right side shows the target pub amongst related distractors church, cafe and kitchen. Pictures are sourced from Wikimedia commons. All images are in the public domain.

We developed a version of the summation task that used pictures rather than words as probes and response options, to establish whether the semantic deficit in RH cases was equivalent across modalities. 40 trials per condition were used to cue a particular location or theme (e.g., pub). Picture probes were chosen which were each weakly related to the concept, but which combined would activate the target (e.g., bell-stool-glass → pub). Each probe was presented one-by-one, with 1 second between presentations. Distractors were related to each probe individually, but not all three together (e.g., pub-church-cafe-kitchen). There were two conditions, shown in Fig. 5: (i) more generic picture probes on a white background that were carefully selected to avoid providing information about the context in which the object would be found, thus maintaining summation demands like the verbal version (referred to as ‘no context’), and (ii) pictures showing more specific objects within the relevant context, which reduced the requirement to summate across images (referred to as ‘with context’).

### 6.4 Results: modality

**Fig. 6:**
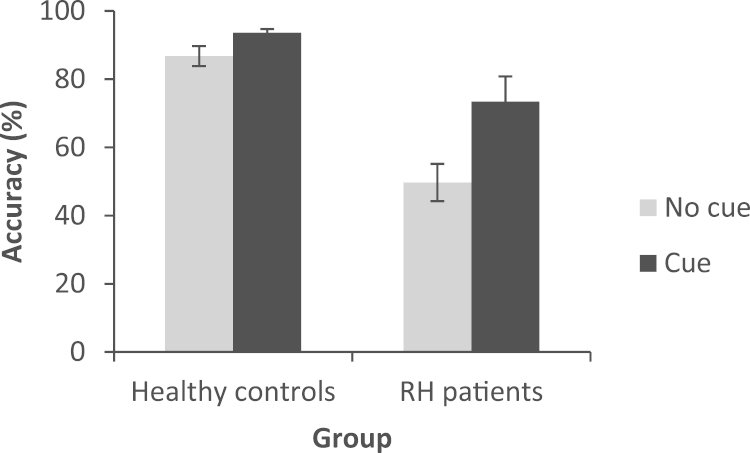
Performance on the picture task in controls and RH patients in each cue condition. Errors bars show standard error of mean.

Data was available for 8 of the RH patients and 10 healthy controls. The results are displayed in Fig. 6. There was a significant main effect of picture context: F(1,16)=42.951, p<.001, group: F(1, 16)=23.907, p<.001, and an interaction of group and contextual cueing: F(1,16)=13.967, p=.002. Both RH patients and controls performed more poorly when picture probes did not provide contextual information, but this effect was larger in RH cases.

We also compared RH patients and controls across modalities using ANOVA. This analysis compared the ‘no cue’ condition in both modalities – where the probe did not constrain semantic retrieval (i.e., the ‘no cue’ verbal condition and the ‘no context’ picture condition). RH patients were impaired at both tasks in relation to controls, leading to a main effect of group: F(1,16)=26.431, p<.001. In this analysis, there was no effect of modality: F(1,16)=2.405, p=.140, but there was a modality by group interaction: F(1,16)=4.787, p=.044. This reflects higher performance for controls in the picture compared with word task (t(9)=2.618, p=.028) but no modality difference in the patients (t<1).

### 6.5 Rationale: distractor type

Next, the distractor type was manipulated, using the verbal version of the task. In the tasks above, the distractors were *strongly related to each of the probes* individually (e.g., cat-attacks-paw → hit-whiskers-foot-scratch). We compared these trials (in the absence of cueing) to a second condition involving distractors that were related to the target word but that were *weakly or unrelated* to the probes (e.g., cat-attacks-paw → scrape-rub-scar-scratch). SA patients have shown strong sensitivity to the strength of distracters in previous studies (Corbett et al., 2014, Noonan et al., 2010). Strong distracters create competition that patients with SA have difficulty resolving appropriately. If RH patients are similarly sensitive to the nature of the distracters, the two groups might show a similar pattern of performance. Alternatively, RH cases might be disproportionately impaired at distracters strongly related to individual probes, compared with SA patients, if they have greater deficits with summation.

### 6.6 Results: distractor type

**Fig. 7:**
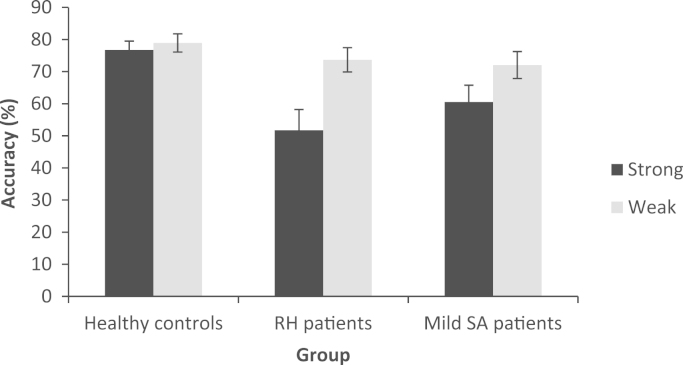
Effect of distractor type on the summation task in controls, RH patients and Mild SA patients. Error bars show standard error of the mean.

Results were available for 6 of the RH patients, 8 mild SA patients, and 12 healthy controls. In a comparison of RH patients and healthy controls, there was a main effect of group: F(1,16)=8.095, p=.012, distractor type: F(1,16)=15.007, p=.001, and an interaction of group and distractor type: F(1,16)=10.279, p=.006. RH patients were similar to healthy controls when distractors were related to the target (t<1.1), but were impaired when distractors were related to individual probes (t(19)=3.914, p=.001). In a comparison of RH and mild SA cases, there was again a main effect of distractor type: F(1,12)=16.743, p=.001, but not of group (F<1), and no interaction: F(1,12)=1.908, p=.192. Both mild SA and RH patients were worse at the task involving distractors strongly related to each probe. This suggests that the two patient groups had similar difficulty suppressing dominant irrelevant meanings from the probe words. This pattern is shown in Fig. 7.

### 6.7 Summary

RH patients showed sensitivity to manipulations of semantic control across modalities. First, like SA cases, they performed poorly when miscues directed retrieval towards irrelevant features of the probes; they also showed positive cueing effects, particularly when the cues reduced the requirement to summate. The pattern of results was similar across modalities. Additionally, both mild SA and RH patients showed an effect of distractor type: they showed a greater impairment in the inhibition of irrelevant items strongly related to individual probe words. This suggests that both patient groups had problems suppressing irrelevant concepts when they were primed. We found that both left and right sided stroke patients were equivalently influenced by distractor strength, in line with the suggestion that bilateral mechanisms are involved in executive control (Duncan, 2010) and that manipulations of semantic control elicit activation in both hemispheres (Noonan et al., 2013). This inability to inhibit irrelevant information has been described across several tasks in SA patients: these patients make associative errors in picture naming that are not relevant to the identity of the item (Jefferies and Lambon Ralph, 2006, Jefferies et al., 2008), occasionally self-cue themselves outside the relevant domain in category fluency (Rogers et al., 2015), and show strong miscueing effects in picture naming and comprehension tasks (Corbett et al., 2011, Noonan et al., 2010). Overall, the data from these experiments point to similarities between SA and RH cases.

## 7 Cyclical matching task

### 7.1 Rationale

SA patients with damage to left-sided semantic control (i.e., left inferior frontal gyrus) regions make increasing numbers of errors in ‘cyclical’ word-picture matching tasks which probe a small set of related concepts repeatedly, such that the same items are presented as targets and distracters on different trials (Gardner et al., 2012, Jefferies et al., 2007, Thompson et al., 2015). This decline across cycles in performance (or ‘refractory’ effect) is thought to follow from a build-up of competition between the items in the set, increasing the demands on selection processes (Campanella and Shallice, 2011, Forde and Humphreys, 1997, Gotts and Plaut, 2002, Warrington and Cipolotti, 1996, Warrington and Crutch, 2004, Warrington and McCarthy, 1983). Importantly, this is not a general fatigue effect, as the decline occurs across cycles within a block, and there is a release from the effect when starting a new block. To our knowledge, studies have not yet examined refractory paradigms in patients with semantic deficits following RH lesions. Since our RH cases were unimpaired on word-picture matching for everyday objects, we investigated the possibility that they would show effects of cycle on a matching task involving facial emotions, since this domain is often impaired after RH damage (Bowers et al., 1991, Harciarek and Heilman, 2009). The blocks were made up of highly competitive, confusable facial emotions. We didn’t run a ‘low competition’ condition here, as in some versions of the task (Jefferies et al., 2007): normally sets of semantically related objects are re-arranged to form semantically unrelated sets but this manipulation cannot be easily achieved for face emotions.

We contrasted word-picture matching (for an emotion word, such as ‘anger’ with an angry face) and picture-picture matching (involving two different angry faces), in order to examine whether any deficits in the RH cases were multimodal. Effects of modality in the cyclical matching task are controversial because there is debate about whether the refractory state occurs (i) within lexical-semantic representations (Harvey and Schnur, 2015), (ii) for auditory tasks specifically (McCarthy and Warrington, 2015), or (iii) whether it reflects competition between amodal semantic representations and thus extends across modalities (Forde and Humphreys, 1997, Gardner et al., 2012). In addition, even if both SA and RH cases show multimodal deficits, the degree of impairment might differ across verbal and non-verbal tasks in the two groups (Krieger-Redwood et al., 2015, Winner and Gardner, 1977).

### 7.2 Procedure

**Fig. 8:**
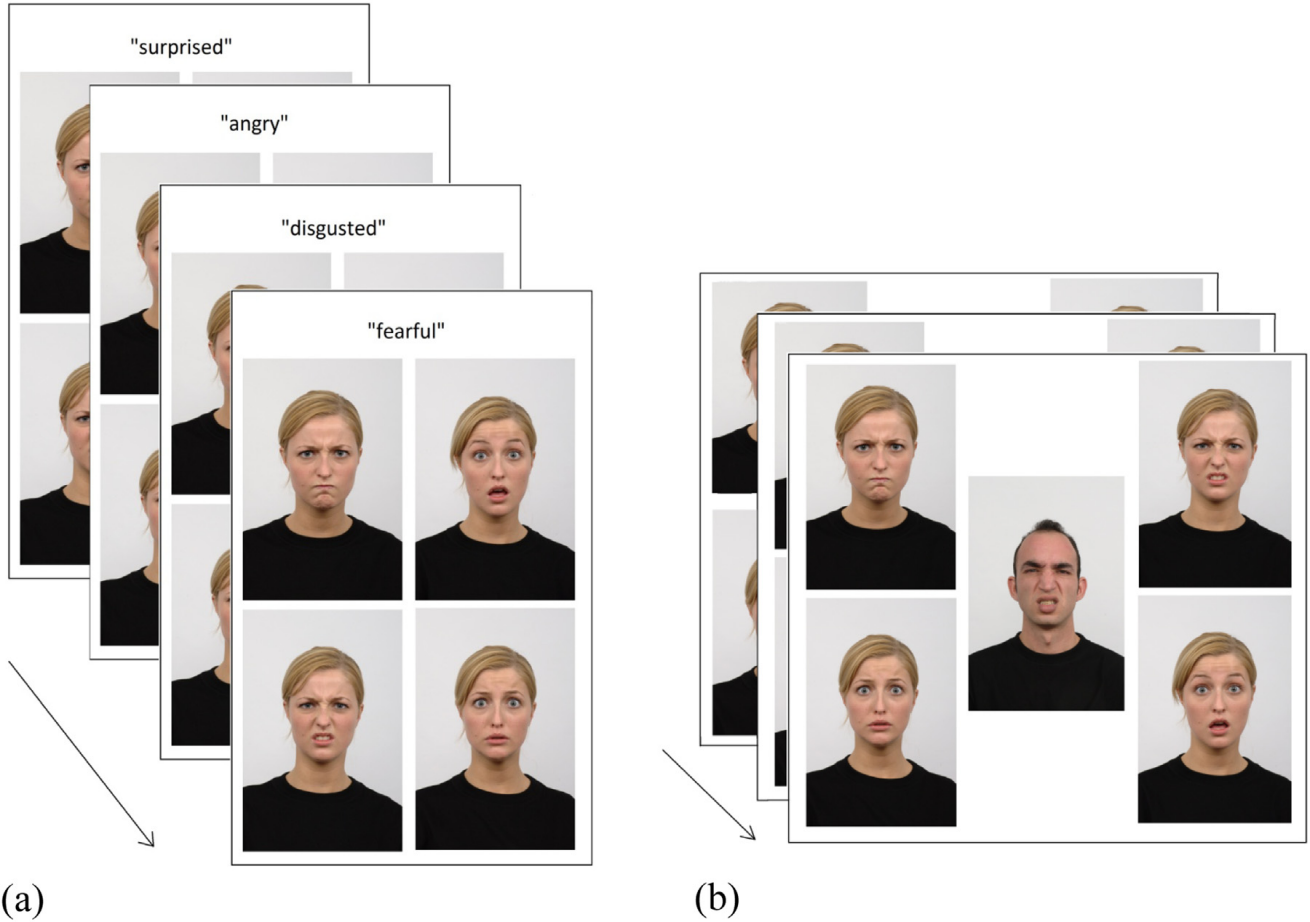
Refractory emotional faces, (a) shows word-picture matching, (b) shows picture-picture matching.

The cyclical emotion matching task involved matching a probe emotion to a target picture of a face with the same emotion. Emotional faces were obtained from the Radboud Faces Database (Langner et al., 2010) and depicted 7 core emotions: happy, sad, angry, surprised, fearful, disgusted and contemptuous. The probe was either a spoken word (word-to-picture matching, WPM) or a picture of another emotional face, presented in the middle of the screen (picture-to-picture matching, PPM). In both tasks, there were four response options (a target and three distractors). Within a block of trials, the same target/distractors were repeatedly probed so that the target face on one trial became the distracter on the next: each item was a target 4 times (in 4 cycles), leading to 16 trials per block. The task is displayed in Fig. 8.

For PPM, the *probe* face was of the opposite gender and a different ethnic group to the target and distractors, to avoid the task being a visual matching paradigm (as much as was possible). For both the WPM and PPM, however, the *target and distractors* were the same identity to maximise the visual overlap between target and distractors. In the WPM task, there were 8 blocks, and in PPM there were 10 blocks. The identity of the faces changed between blocks. Participants indicated their responses by pointing; the researcher then immediately pressed a button to advance the experiment and recorded accuracy of the decision. After 10 s without a response, the next trial was presented. Accuracy was the dependent variable.

### 7.3 Results

**Fig. 9:**
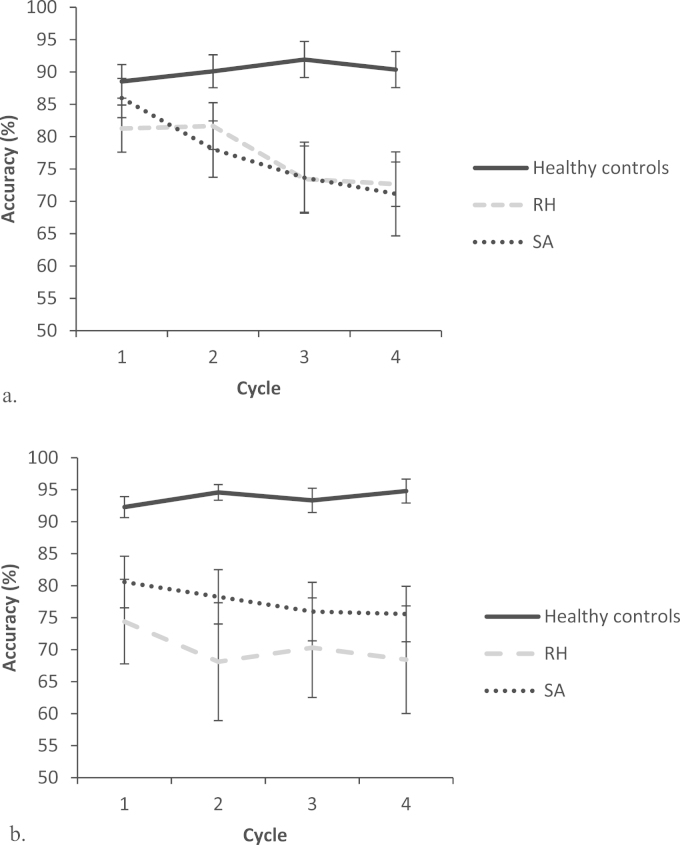
Effect of cycle on accuracy for SA patients, healthy controls and RH patients. SA patients performed an object matching task (Thompson et al., 2015, Gardner et al., 2012), whereas RH patients and controls performed an emotional face matching task. a. shows word-picture matching, b. shows picture-picture matching. Error bars show standard error of mean.

In a comparison of RH patients and controls, there was a main effect of cycle: F(3,16)=3.527, p=.039, a main effect of group: F(1,18)=15.921, p=.001, and a group by cycle interaction: F(3,16)=9.152, p=.001. There was no main effect of modality (F<1), no interaction between modality and cycle (F<1.3) nor between modality and group (F(1,18)=2.803, p=.111). There was a three-way interaction between modality, cycle and group: F(3,16)=3.717, p=.033, reflecting higher performance in PPM than WPM in controls, and lower performance in PPM than WPM in RH patients. There were also larger effects of cycle in the WPM than PPM. Over the experiment, controls showed an increase in performance across cycles, and RH patients showed a decrease, and this pattern is accentuated in WPM compared to the PPM. This pattern is shown in Fig. 9.

### 7.4 Comparison with SA patients

Cyclical effects in RH and SA cases were compared using data from 13 SA patients reported by Thompson et al. (2015). SA patients were examined on cyclical WPM and PPM tasks for sets of semantically-related common objects (e.g., fork, spoon, spatula, or knife), allowing us to compare effects of cycle in SA and RH patients on two tasks that were broadly matched for accuracy. The groups showed a similar pattern of performance (Fig. 9). There was a main effect of cycle: F(3,17)=4.792, p=.013, but no effect of group (F<1) or modality (F<1.1). There was a modality by cycle interaction: F(3,17)=4.310, p=.020, reflecting greater decline in accuracy in WPM than PPM. This was true in both groups: there was no three-way interaction: F(3,17)=1.864, p=.174. There was also no interaction of modality and group, or cycle and group (F<1.4).

### 7.5 Summary

For the first time, RH damage has been shown to elicit ‘refractory effects’, or increasingly poor performance across cycles. This pattern of declining accuracy in RH patients for face emotion tasks was similar to that previously reported for SA cases in matching tasks using sets of semantically-related common objects. These findings are consistent with the hypothesis that both groups have poor executive control over semantic retrieval and thus have increased difficulty when the distracters were previously targets (and are therefore primed), and when distracters have to be re-selected as targets after they have been inhibited.

## 8 Discussion

This case-series study characterises the semantic control deficits arising from right hemisphere (RH) brain damage following stroke and compares this pattern of impairment to semantic aphasia (SA) which is characterised by deficient semantic control following left-hemisphere stroke. Neuroimaging studies have previously highlighted the contribution of a left-dominant yet bilateral network to controlled semantic retrieval (Noonan et al., 2013). In addition, neuropsychological investigations of patients with SA have confirmed that lesions to left IFG and/or left temporoparietal cortex give rise to the inappropriate retrieval of dominant yet irrelevant knowledge, plus difficulty retrieving relevant information in the absence of external constraints or when there is strong competition. Although RH cases can also have semantic deficits, the contribution of this hemisphere to *semantic control* has rarely been studied. To address this gap in the literature, we presented semantic tasks designed to be sensitive to RH damage, which simultaneously manipulated the requirement for controlled retrieval. Under these circumstances, RH patients showed deficits of semantic control that were qualitatively similar to SA patients with left-sided lesions. Below, we summarise our key findings and then present a theoretical perspective that can accommodate these data.

1. We found that RH and SA cases had strikingly different levels of impairment in standard assessments of comprehension: RH patients performed at a normal level on every task in our battery, while SA cases (even mild SA cases) showed significant deficits. This suggests a marked *quantitative* difference between patients with left- and right-sided lesions in the degree of semantic impairment, consistent with functional neuroimaging studies of semantic control that show substantially more left- than right-sided recruitment (e.g., Noonan et al., 2013).
2. There were also subtle *qualitative* differences between RH and SA cases. RH cases were not impaired at a demanding “semantic buffer” task, which involved holding in mind several words and making semantic judgements about them individually – even though this task was highly sensitive to the deficits in mild SA (Hoffman et al., 2009, Hoffman et al., 2011). Yet in a ‘summation’ task, RH cases had greater difficulty than would be expected from their performance on standard semantic assessments. This paradigm required patients to integrate the meanings of several items to identify a distantly-related target. In addition, RH cases showed poorer understanding of non-literal meanings, especially when these were presented as pictures, and they were more impaired in a sentence completion task, when they were asked to avoid producing the predicted ending strongly primed by the sentence but instead to say something unconnected. These findings are largely consistent with previous proposals that have argued for a particular contribution of the right hemisphere to (i) broad as opposed to narrow semantic fields (Beeman, 1998, Beeman et al., 1994, Blake et al., 2015, Jung-Beeman, 2005) and (ii) within the domain of executive control, inhibition as opposed to selection (Aron et al., 2014, Chikazoe et al., 2007, Lenartowicz et al., 2011, Levy and Wagner, 2011).
3. Despite these quantitative and qualitative differences between RH and SA patients, there were *similarities* between the groups in terms of the effects of semantic control demands on comprehension. Previous work has found that SA patients with left-sided damage have better semantic retrieval when cues are provided – and poorer performance with miscues (Jefferies et al., 2008, Soni et al., 2009). They also show an inability to inhibit prepotent distractors (Noonan et al., 2010) and a decline in accuracy in cyclical matching tasks as sets of items are repeated (Gardner et al., 2012, Jefferies et al., 2007). These findings suggest that SA patients do not have damage to a central store of semantic information, but rather difficulty retrieving information from this store in an appropriate fashion. This study documented similar phenomena in the RH group, using semantic tasks sensitive to RH lesions (i.e., summation and face emotion matching tasks). On a summation task, both groups showed improved comprehension with cues and poorer semantic retrieval with miscues. The two groups also showed equivalent effects of distracter type – namely, poorer performance when distracters were related to the cue words as opposed to the target word. Finally, in a cyclical matching paradigm, both groups showed deteriorating performance when the same concepts were probed repeatedly such that the targets became distractors (requiring inhibition) and the distracters became targets (increasing selection requirements).

These similarities between SA and RH cases suggest that, in *both* groups, there is a failure of executive-semantic processing. Our RH cases had lesions broadly similar to those that characterise SA in the opposite hemisphere. In both groups, the damage is typically centred on posterior frontal and temporoparietal areas (Gardner et al., 2012, Jefferies, 2013, Noonan et al., 2010), and crucially spares the ventral ATL which is the focus of atrophy in semantic dementia (Binney et al., 2010, Hoffman et al., 2012). Ventral ATL is argued to underpin a key store of amodal semantic knowledge (Binney et al., 2012, Visser et al., 2012) and is a watershed region rarely damaged in stroke (Phan et al., 2005, Phan et al., 2007). Instead, the posterior frontal and temporoparietal areas that were commonly damaged in our SA and RH cases have been linked to the selection and controlled retrieval of semantic knowledge (Badre et al., 2005, Noonan et al., 2013, Thompson-Schill et al., 1997). This is likely to explain the inconsistent retrieval of information and strong sensitivity to task demands in both groups. Cues reduce executive demands since they pre-activate task-relevant features and reduce competition with the target concept. Similarly, miscues pre-activate irrelevant features and amplify competition, increasing the need to engage executive mechanisms. The decline in performance seen in both groups in the cyclical matching paradigm can be understood in a similar way. On later cycles, all of the items in the set were highly active creating a high level of competition and interfering with the selection of the target and the inhibition of distractors in both SA and RH patients. Difficulties on this task have been specifically linked to lexical/semantic selection deficits following damage to left IFG in previous work (Campanella et al., 2012, Campanella et al., 2009, Gardner et al., 2012, Harvey and Schnur, 2015, Jefferies et al., 2007, Schnur et al., 2006, Schnur et al., 2009). Our novel observation of this pattern in RH patients suggests that this conclusion should be extended to encompass brain regions in the opposite hemisphere. Thus, the primary deficit in comprehension-impaired stroke patients with left and right hemisphere lesions appears not to be a loss of knowledge from the semantic store per se, but difficulty controlling retrieval of this knowledge appropriately in the absence of external support.

Although the central focus of our investigation was to document the parallel impairment of semantic control in SA and RH patients, there were striking quantitative and qualitative differences in their semantic deficits which meant this issue could only be investigated using semantic tasks designed to be maximally sensitive to RH damage. The remainder of this discussion considers explanations for these differences in the context of the similar semantic control deficit. First, we discuss the effects of modality. Patients with SA have parallel deficits of semantic control in verbal and non-verbal tasks, consistent with damage to modality-general control mechanisms that interact with amodal semantic representations in intact ventral ATL (Corbett et al., 2009, Corbett et al., 2011, Jefferies and Lambon Ralph, 2006, Rogers et al., 2015). SA patients show parallel effects of cycle in word-picture and picture-picture matching tasks, they have disordered semantic retrieval in purely non-verbal domains such as object use, and they show similar effects of cueing manipulations and distractor strength in verbal and non-verbal tasks (Corbett et al., 2011, Jefferies et al., 2008, Noonan et al., 2010). A bilateral semantic control network, which partially overlaps with the ‘multi-demand’ executive system, may be recruited to support control-demanding semantic tasks across modalities (Duncan, 2010, Duncan and Owen, 2000). However, previous studies of patients with right-sided stroke have emphasised their semantic deficits on non-verbal tasks – for example, they show poorer performance with picture than word metaphor comprehension (Winner and Gardner, 1977). We included several contrasts between verbal and non-verbal tasks in this study. In line with existing findings, our RH patients were more impaired at a picture metaphor task, even though they performed similarly to mild SA cases on a word metaphor version. RH cases also performed more poorly when matching two faces depicting the same emotion compared with matching a spoken emotional label with a face. However, in the cyclical matching task, the decline over cycles was equivalent for the verbal and non-verbal conditions, and patients showed equivalent performance for picture and word versions of the summation task. Therefore, it seems that RH patients, like those with SA, have deficient semantic control across modalities (even though modality effects in the RH group differ to some extent across tasks).

The RH cases were largely unimpaired at both verbal and non-verbal semantic tasks requiring comprehension of individual items without summation, even challenging tasks like the ‘semantic buffer’ task. Thus, despite evidence for a role of both left and right hemispheres in controlled semantic retrieval across modalities, there might be some important differences in the contribution of the two hemispheres to semantic processing. For example, the RH might make a stronger contribution to ‘holistic’ processing – required in both the summation task (Beeman et al., 1994, Jung-Beeman, 2005) and for recognising facial emotions (Calder et al., 2000, Flack et al., 2015, Maurer et al., 2002). This possibility is broadly consistent with the Bilateral Activation, Integration and Selection hypothesis (Jung-Beeman, 2005), which suggests the RH uses relatively coarse semantic coding, while the LH uses finer coding. The LH might be focused on selecting features which are dominant or contextually relevant, whilst inhibiting alternatives. Consequently, SA patients with LH damage would have difficulty focussing their retrieval on relevant information. In contrast, the RH might maintain weak, diffuse activation of a broader semantic field, including distant and unusual semantic features and associations, and information that fits poorly with the context. This would allow the RH to summate weakly connected meanings and could explain the deficits of RH cases on the summation task.

Within our account of semantic cognition, these differences are unlikely to arise from hemispheric differences within the ATL ‘semantic store’, since a recent review indicates that while the left ATL shows greater engagement for semantic tasks involving speech production, the degree of specialisation within this system is relatively subtle (Rice et al., 2015). Hemispheric specialisation is also unlikely to reflect strong differences in executive control processes, since we have demonstrated that the two groups had broadly parallel problems in resolving competition between concepts. One possibility is that while a multi-demand executive system supports demanding semantic judgements in both the left and right hemisphere, this system is partially separable from regions that support semantic control – e.g., in anterior/ventral IFG and pMTG – and that these semantic but not domain-general executive control processes are left-lateralised (cf. Noonan et al., 2013). This hypothesis can explain why our left-sided SA cases and RH patients showed largely equivalent executive control deficits and yet the SA patients showed a much greater degree of impairment on simple semantic tasks. The regions damaged in SA would tend to affect structures implicated in domain-general executive control (e.g., inferior frontal sulcus) as well as regions exclusively engaged by control-demanding semantic judgements, such as pMTG and ventral/anterior IFG. Damage to both of these neurocognitive components would result in severe impairment on control-demanding semantic tasks, even those involving single items and/or relatively simple judgements. In contrast, RH damage would impair domain-general executive processing but leave left-sided semantic control processes intact: this would be expected to result in sensitivity to the control demands of semantic tasks but stronger semantic performance overall.

The specialisation of the left hemisphere for semantic control might explain its greater contribution to the selection of specific concepts and words one at a time (e.g., for speech production; Schnur et al., 2005, Schwartz et al., 2006). Importantly, given the similarities between verbal and non-verbal tasks discussed above, there is evidence for a role of the LH in the retrieval of specific/narrow conceptual information beyond speech production – for example, the network of brain regions implicated in action understanding is also strongly left-lateralised (Davey et al., 2015, Kemmerer et al., 2012, Liljestrom et al., 2008, Watson et al., 2013).

In conclusion, this study presents the first case-series comparison of (i) SA patients with deregulated semantic retrieval following left hemisphere lesions and (ii) RH patients selected to show some degree of semantic/language deficit. We found that, like SA patients, our RH cases showed evidence of deficient semantic control such that the likelihood of successful retrieval varied according to: the availability of cues, the presence of miscues, the nature of distracters, and the repetition of items within a cyclical matching task. Thus, patients with both left and right hemisphere stroke have problems with the controlled retrieval of semantic information, and appear to retain knowledge that they are nevertheless unable to retrieve under certain circumstances. This might reflect the fact that stroke typically spares ventral ATL (thought to provide a key store of amodal semantic information) while damage is centred on posterior inferior frontal and temporoparietal structures involved in the controlled retrieval and selection of this information. This similarity between the two groups was observed in the context of other pronounced differences between SA and RH cases reflecting a greater contribution of the left hemisphere to semantic control.

## Figures and Tables

**Table 1:** Demographics of RH patients.

	Age	Gender	Education (age left education)	Years since CVA	PF lesion	TP lesion
ASW	50	F	21	2	✓	✗
SYN	73	M	16	7	✓	✓
DNQ	66	M	16	2	✓	✓
DGX	85	M	16	5	✗	✓
ESP	85	F	14	11	?	?
RHE	56	M	14	3	?	?
DSW	67	M	17	3	✗	✓
BWJ	73	M	15	4	?	?
NDW	68	M	15	6	✓	✓
Average mild SA	57.3 (11.2)	5/20 females	16.2 (1.3)	7.1 (4.6)		
Average SA	69.8 (13.7)	5/11 females	15.6 (1.1)	5.7 (5.2)		
Average RH	69.2 (11.7)	2/9 females	16.0 (2.1)	4.8 (2.9)		

**Table 2:** Lesion analysis for patients.

Patient	Lesion size (% of template damaged)	Group		DLPFC	orbIFG	trIFG	opIFG	PMC	STG	MTG	ITG	FG	POT	AG	SMG	sTP	OL
			BA9	BA46	BA47	BA45	BA44	BA6	BA22	BA21	BA20	BA36	BA37	BA39	BA40	BA38	BA19
ASW	4	RH	–	–	–	2	2	2	1	–	–	–	–	–	–	–	–
SYN	7	RH	–	–	–	1	–	2	2	1	–	–	2	1	2	–	–
DNQ	11	RH	–	–	–	–	2	1	2	2	–	–	2	2	2	–	–
DGX	3	RH	–	–	–	–	–	1	1	1	–	–	–	–	–	–	–
NDW	8	RH	–	–	–	–	1	1	2	–	–	–	1	–	–	–	–
NGW	8	Mild SA	–	–	–	–	1	2	2	1	–	–	2	–	1	–	–
SSR	15	Mild SA	–	–	2	1	2	2	2	–	–	–	1	1	1	–	–
RTJ	14	Mild SA	–	1	–	2	2	2	2	–	–	–	–	–	2	–	–
NHY	7	Mild SA	–	–	–	1	2	1	–	–	–	–	1	1	1	–	–
ESU	15	Mild SA	–	–	–	–	2	2	1	2	–	–	1	1	2	–	–
NNZ	4	Mild SA	–	–	–	–	1	1	1	1	–	–	1	–	–	–	–
YHE	9	Mild SA	–	–	–	1	2	–	2	–	–	–	–	–	–	–	–
Average RH			0%	0%	0%	40%	60%	100%	100%	60%	0%	0%	60%	40%	40%	0%	0%
Average mild SA			0%	14%	14%	57%	100%	86%	86%	43%	0%	0%	71%	43%	71%	0%	0%
Average SA			33%	27%	47%	53%	67%	60%	53%	53%	40%	13%	73%	53%	60%	13%	40%

Lesions in RH patients are in the right hemisphere, and mild SA in the left hemisphere. SA patients shown as percentage of total with lesion to each regions, full details of the majority (13/15) SA patients have been previously described (Gardner et al., 2012, Thompson et al., 2015). The percentages are also given for RH and mild SA cases to aid comparison between groups. Missing from the table are RH patients: ESP, RHE, DSW and BWJ; and mild SA cases: JHU, JB, ABU, and ONY. These patients were either never scanned, or provided radiographers reports which are described above. Quantification of lesion: 2=complete destruction/serious damage to cortical grey matter; 1=partial destruction/mild damage to cortical grey matter. Anatomical abbreviations: DLPFC=dorsolateral prefrontal cortex; orbIFG=pars orbitalis in inferior frontal gyrus; trIFG,=pars triangularis in inferior frontal gyrus; opIFG=pars opercularis in inferior frontal gyrus; PMC=premotor cortex; STG=superior temporal gyrus; MTG=middle temporal gyrus; ITG=inferior temporal gyrus; FG=fusiform gyrus; POT=posterior occipitotemporal area; AG=angular gyrus; SMG=supramarginal gyrus; sTP=superior pole; OL=occipital lobe.

**Table 3:** Background scores for language and semantic tasks in RH cases.

	Right Hemisphere Language Battery						FANL		CCT	Synonym						Fluency	
	Metaphor pictures	Metaphor words	Lexical semantic (WPM)	Inferences	Humour test	Emphatic stress	Metaphors (pictures)	Literal phrases (pictures)	CCT pictures	Synonym task-total	Low imageability	Medium imageability	High imageability	Low frequency	High frequency	Category Fluency	Letter Fluency
Max	10	10	20	12	10	10	20	20	*64*	*96*	*32*	*32*	*32*	*48*	*48*	NA	NA
Normal cut off	8.3	8.3	19.4	8	8.8	10	16.7	16.6	52	91	27.6	30.8	30.9	44.9	44.4	8.7 b	8.3 b
*Average SA*									39.5^a^	67.9^a^	17^a^	22.5^a^	27.6^a^	33.2^a^	34^a^	4.7^a^	2.2^a^
*Average mild-SA*	7.6^a^	9.1	19.3^a^	9.3	6.4^a^	7.3^a^	15.0^a^	14.9^a^	55.6	78.4^a^	18.3^a^	28.9^a^	30.4^a^	38.6^a^	39.0^a^	11.4	3.9^a^
*Average RH*	4.9^a^	9.8	19.2^a^	10.0	6.1^a^	6.2^a^	15.7^a^	17.8	55	89^a^	26^a^	32	31	45	44^a^	18	12
																	
*SYN*	10	10	18^a^	12	5^a^	9^a^	13^a^	16^a^	52	95	31	32	32	48	47	16	8^a^
*ASW*	6^a^	10	19^a^	9	9	8^a^	16^a^	19	56	88^a^	29	30^a^	29^a^	45	43^a^	18	17
*DNQ*	6^a^	10	20	10	0^a^	5^a^	15^a^	19	55	89^a^	26^a^	32	31	44^a^	45	21	12
*DGX*	0^a^	10	19^a^	11	9	6^a^	17^a^	18	57	94	30	32	32	48	46	13	12
*RHE*	3^a^	9	19^a^	9	3^a^	4^a^	16^a^	19	58	79^a^	16^a^	32	31	38^a^	41^a^	18	18
*ESP*	6^a^	10	18^a^	12	7^a^	7^a^	16^a^	19	54	87^a^	24^a^	31	32	45	42^a^	12	12
*DSW*	5^a^	10	20	11	6^a^	8^a^	19	20	61	93	30	32	31	46	46	18	6^a^
*NDW*	2^a^	10	20	10	8^a^	5^a^	15^a^	14^a^	55	90^a^	27^a^	32	31	46	44	23	14
*BWJ*	6^a^	10	20	6^a^	8^a^	6^a^	14^a^	16^a^	49^a^	87^a^	25^a^	31	31	44	43^a^	19	7^a^

Right hemisphere language tasks taken from the Right Hemisphere Language Battery (Bryan, 1995). WPM=spoken word to picture matching. FANL=Familiar and Novel Language Comprehension Test (Kempler and Van Lancker, 1985). CCT=camel and cactus test of associative semantic knowledge (Bozeat et al., 2000). Synonym=96 item synonym matching task (Jefferies et al., 2009). Category and letter fluency (letter=‘S’, category=animals). Number of patients populating averages for SA and mild SA are as follows: metaphor pictures and words (SA=NT, mild SA=8), inference (SA=NT, mild SA=6), humour (SA=NT, mild SA=7), emphatic stress (SA=NT, mild SA=3), metaphor and literal phrases-FANL (SA=NT, mild SA=8), CCT pictures (SA=19, mild SA=11), synonym task (SA=20, mild SA=7), category fluency (SA=18, mild SA=7), letter fluency (SA=17, mild SA=7). NT = not tested.

a Impaired below normal cut-off. Control performance and normal cut-offs taken from published texts except where stated.

b Norms from 15 healthy controls tested at the University of York, cut off 2 SD below the mean.

**Table 4:** Background scores for visual and executive tasks in RH cases.

	VOSP									Bells				Hayling			TEA		Trails		Digit span	
	Screening	Incomplete letters	Silhouettes	Object decision	Progressive silhouettes	Dot counting	Position	Number location	Cube analysis	Left half	Right half	Difference^e^	BSRA	Sensible	Unconnected	RCPM (A, AB, B)	TEA	TEA (with distraction)	Trail making (A)	Trail making (B)	Digit Span forwards	Digit Span backwards
Max	*20*	*20*	*30*	*20*	*–*	*10*	*20*	*10*	*10*	18	17		*54*	*15*	*15*	*36*	*7*	*10*	*24*	*23*	*8*	*7*
Normal cut off	19.3	16.9	10	10.5	6.0^d^	9.5	17.1	4.7	5.4				28	11	11	28^b^	6	3	24^c^	17.4^c^	5	2
*Average SA*	19.5	11.4^a^		15.8	6.9^a^	7.5^a^	16.2^a^	8.2	5.3^a^	–	–	–	20.9^a^	11.5	9.8^a^	23.5^a^	4.7^a^	3.6	21^a^	10.1^a^	4^a^	1.7^a^
*Average mild SA*		19.3				9.5	19.2	9.1	8.6	–	–	–	32.1	–	–	27.9^a^	5.2^a^	2.4^a^	23.9^a^	15.8^a^	5.2	2.8
*Average RH*	19^a^	19	20	17	12^a^	10	20	9	7	16	15	1	27.2^a^	10.8^a^	1.5^a^	27.2^a^	6.6	4.6	24	17^a^	5.8	3.1
																						
*SYN*	18^a^	18	13	16	15^a^	9^a^	19	7	4^a^	16	16	0	38	14	1^a^	26^a^	6	0^a^	24	7^a^	4^a^	3
*ASW*	20	20	24	18	10^a^	10	20	10	10	18	17	1	21^a^	14	6^a^	30	6	9	24	22	7	4
*DNQ*	19^a^	19	21	18	10^a^	10	20	5	8	13	14	1	31	15	7^a^	24^a^	6	5	24	23	6	3
*DGX*	18^a^	20	17	14	13^a^	8^a^	20	10	9	17	17	0	27^a^	12	1^a^	35	7	10	24	23	5	2
*RHE*	20	20	23	19	11^a^	10	19	10	7	18	16	2	27^a^	13	1^a^	23^a^	7	2^a^	24	17^a^	4^a^	2
*ESP*	17^a^	19	15	15	10^a^	10	20	9	3^a^	14	12	2	24^a^	13	5^a^	21^a^	6	7	24	17^a^	5	2
*DSW*	20	19	22	18	15^a^	10	17^a^	10	10	18	17	1	39	14	1^a^	32	7	1^a^	24	20	6	4
*NDW*	20	18	17	15	12^a^	10	18	9	9	16	15	1	29	15	4^a^	31	7	5	24	17^a^	8	6
*BWJ*	19^a^	16^a^	19	14	12^a^	10	17^a^	6	6	15	13	2	9^a^	13	0^a^	23^a^	7	2^a^	24	17^a^	7	2

VOSP=visual object and space processing battery (Warrington and James, 1991); Bells Cancellation test (Gauthier, Dehaut and Joanette, 1989); RCPM=Raven's Coloured Progressive Matrices (Raven, 1962); BSRA=Brixton spatial rule attainment task (Burgess and Shallice, 1997); TEA=elevator counting with and without distraction from the test of everyday attention (Robertson et al., 1994). number of SA and mild SA cases populating averages are as follows: VOSP screening (SA=10, mild SA=NT), incomplete letters (SA=8, mild SA=3), silhouettes (SA/mild SA=NT), object decision (SA=9, mild SA=NT), progressive silhouettes (SA=8, mild SA=NT), dot counting (SA=18, mild SA=6), position discrimination (SA=18, mild SA=6), number location (SA=18, mild SA=9, cube analysis (SA=18, mild SA=9), bells cancellation test (SA/mild SA=NT), BSRA (SA=19, mild SA=11), Hayling sensible (SA=11), Hayling unconnected (SA=10), RCPM (SA=20, mild SA=8), TEA with/without distraction (SA=18, mild SA=5), Trail making A/B (SA=9, mild SA=8), digit span forwards (SA=18, mild SA=10), digit span backwards (SA=18, mild SA=9). NT = not tested.

a Impaired. Control performance and normal cut-offs taken from published texts except where stated.

b Norms from 20 healthy controls tested at the University of York.

c Accuracy norms from 14 healthy controls tested at the University of York.

d Anything above this number is impaired.

e The difference between the score for the left and right halves of the page on the Bells cancellation task.

## Footnotes

Appendix A Supplementary data associated with this article can be found in the online version at http://dx.doi.org/10.1016/j.neuropsychologia.2016.02.030.

## References

[bib1] Adolphs R. (2002). Neural systems for Recognizing emotion. Curr. Opin. Neurobiol..

[bib2] Almaghyuli A., Thompson H.E., Lambon Ralph M.A., Jefferies E. (2012). Deficits of semantic control produce absent or reverse frequency effects in comprehension: evidence from neuropsychology and dual task methodology. Neuropsychologia.

[bib3] Anaki D., Faust M., Kravetz S. (1998). Cerebral hemispheric asymmetries in processing lexical metaphors. Neuropsychologia.

[bib4] Aron A.R., Robbins T.W., Poldrack R.A. (2014). Inhibition and the right inferior frontal cortex: one decade on. Trends Cognit. Sci..

[bib5] Badre D., Poldrack R.A., Paré-Blagoev E.J., Insler R.Z., Wagner A.D. (2005). Dissociable controlled retrieval and generalized selection mechanisms in Ventrolateral prefrontal cortex. Neuron.

[bib6] Badre D., Wagner A.D. (2007). Left Ventrolateral prefrontal cortex and the cognitive control of memory. Neuropsychologia.

[bib7] Beeman M., Beeman M., Chiarello C. (1998). Coarse semantic coding and discourse comprehension. Right Hemisphere Language Comprehension: Perspective from Cognitive Neuroscience.

[bib8] Beeman M., Friedman R.B., Grafman J., Perez E., Diamond S., Beadle Lindsay M. (1994). Summation priming and coarse semantic coding in the right hemisphere. J. Cognit. Neurosci..

[bib9] Binney R.J., Embleton K.V., Jefferies E., Parker G.J.M., Lambon Ralph M.A. (2010). The ventral and Inferolateral aspects of the anterior temporal lobe are crucial in semantic memory: evidence from a novel direct comparison of distortion-corrected fMRI, rTMS, and semantic dementia. Cereb. Cortex.

[bib10] Binney R.J., Parker G.J.M., Lambon Ralph M.A. (2012). Convergent connectivity and graded specialization in the rostral human temporal lobe as revealed by diffusion-weighted imaging probabilistic Tractography. J. Cognit. Neurosci..

[bib11] Blake M.L., Tompkins C.A., Scharp V.L., Meigh K., Wambaugh J. (2015). Contextual constraint treatment for coarse coding deficit in adults with right hemisphere brain damage: generalisation to narrative discourse comprehension. Neuropsychol. Rehabil..

[bib12] Blonder L.X., Bowers D., Heilman K.M. (1991). The role of the right hemisphere in emotional communication. Brain.

[bib13] Borden N.M. (2006). 3D Angiographic Atlas of Neurovascular Anatomy and Pathology.

[bib15] Bowers D., Blonder L.X., Feinberg T., Heilman K.M. (1991). Differential impact of right and left hemisphere lesions on facial emotion and object imagery. Brain.

[bib16] Bozeat S., Lambon Ralph M.A., Patterson K., Garrard P., Hodges J.R. (2000). Non-verbal semantic impairment in semantic dementia. Neuropsychologia.

[bib17] Brownell H.H., Simpson T.L., Bihrle A.M., Potter H.H., Gardner H. (1990). Appreciation of metaphoric alternative word meanings by left and right brain-damaged patients. Neuropsychologia.

[bib18] Bryan K.L. (1995). The Right Hemisphere Language Battery.

[bib19] Burgess P.W., Shallice T. (1997). The Hayling and Brixton Tests.

[bib20] Calder A.J., Young A.W., Keane J., Dean M. (2000). Configural information in facial expression perception. J. Exp. Psychology: Hum. Percept. Perform..

[bib21] Campanella F., Crescentini C., Mussoni A., Skrap M. (2012). Refractory semantic access dysphasia resulting from resection of a left frontal glioma. Neurocase.

[bib22] Campanella F., Mondani M., Skrap M., Shallice T. (2009). Semantic access dysphasia resulting from left temporal lobe tumours. Brain.

[bib23] Campanella F., Shallice T. (2011). Refractoriness and the healthy brain: a behavioural study on semantic access. Cognition.

[bib24] Chechlacz, M., Mantini, D., Gillebert, C. R., & Humphreys, G. W., Asymmetrical white matter networks for attending to global versus local features. Cortex, 72, 2015, 54-64. 10.1016/j.cortex.2015.01.022 PMC4643681 25727548

[bib25] Chen E., Widick P., Chatterjee A. (2008). Functional-anatomical organization of predicate metaphor processing. Brain Lang..

[bib26] Chikazoe J., Konishi S., Asari T., Jimura K., Miyashita Y. (2007). Activation of right inferior frontal gyrus during response inhibition across response modalities. J. Cognit. Neurosci..

[bib27] Conn P.M. (2003). Neuroscience in Medicine.

[bib28] Corbett F., Jefferies E., Burns A., Lambon Ralph M.A. (2014). Deregulated semantic cognition contributes to object-use deficits in Alzheimer’s disease: a comparison with semantic aphasia and semantic dementia. J. Neuropsychol..

[bib29] Corbett F., Jefferies E., Ehsan S., Lambon Ralph M.A. (2009). Different impairments of semantic cognition in semantic dementia and semantic aphasia: evidence from the non-verbal domain. Brain.

[bib30] Corbett F., Jefferies E., Lambon Ralph M.A. (2008). The use of cueing to alleviate recurrent verbal perseverations: evidence from Transcortical sensory aphasia. Aphasiology.

[bib31] Corbett F., Jefferies E., Lambon Ralph M.A. (2009). Exploring Multimodal semantic control impairments in semantic aphasia: evidence from naturalistic object use. Neuropsychologia.

[bib32] Corbett F., Jefferies E., Lambon Ralph M.A. (2011). Deregulated semantic cognition follows prefrontal and Temporoparietal damage: evidence from the impact of task constraint on non-verbal object use. J. Cognit. Neurosci..

[bib33] Crawford J.R., Garthwaite P.H., Porter S. (2010). Point and interval estimates of effect sizes for the case-controls design in neuropsychology: rationale, methods, implementations, and proposed reporting standards. Cognit. Neuropsychol..

[bib34] Damasio H., Damasio A.R. (1989). Lesion Analysis in Neuropsychology.

[bib35] Davey J., Rueschemeyer S.A., Costigan A., Murphy N., Krieger-Redwood K., Hallam G., Jefferies E. (2015). Shared neural processes support semantic control and action understanding. Brain Lang..

[bib36] Dien J. (2009). A tale of two recognition systems: implications of the fusiform face area and the visual word form area for lateralized object recognition models. Neuropsychologia.

[bib37] Duncan J. (2006). EPS mid-career award 2004: brain mechanisms of attention. Q. J. Exp. Psychol..

[bib38] Duncan J. (2010). The multiple-demand (MD) system of the Primate brain: mental programs for intelligent behaviour. Trends Cognit. Sci..

[bib39] Duncan J., Owen A.M. (2000). Common regions of the human frontal lobe recruited by diverse cognitive demands. Trends Neurosci..

[bib40] Erika-Florence M., Leech R., Hampshire A. (2014). A functional network perspective on response inhibition and attentional control. Nat. Commun..

[bib41] Faust M., Mashal N. (2007). The role of the right cerebral hemisphere in processing novel metaphoric expressions taken from poetry: a divided visual field study. Neuropsychologia.

[bib42] Flack T.R., Andrews T.J., Hymers M., Al-Mosaiwi M., Marsden S.P., Strachan J.W.A., Trakulpipat C., Wang L., Wu T., Young A.W. (2015). Responses in the right posterior superior temporal sulcus show a feature-based response to facial expression. Cortex.

[bib43] Foldi N.S., Cicone M., Gardner H., Segalowitz S.J. (1983). Pragmatic aspects of communications in brain damaged patients. Language Functions and Brain Organization.

[bib44] Folstein M.F., Folstein S.E., McHugh P.R. (1975). Mini-mental state: a practical method of grading the cognitive state of patients for the clinician. J. Psychiatr. Res..

[bib45] Forde E.M.E., Humphreys G.W. (1997). A semantic locus for refractory behaviour: implications for access storage distinctions and the nature of semantic memory. Cognit. Neuropsychol..

[bib46] Gagnon L., Goulet P., Giroux F., Joanette Y. (2003). Processing of metaphoric and non-metaphoric alternative meanings of Words after right- and left-hemispheric lesion. Brain Lang..

[bib47] Galton C.J., Patterson K., Graham K.S., Lambon Ralph M.A. (2001). Differing patterns of temporal atrophy in Alzheimer’s disease and semantic dementia. Neurology.

[bib48] Gardner H.E., Lambon Ralph M.A., Dodds N., Jones T., Eshan S., Jefferies E. (2012). The differential contributions of pFC and Temporoparietal cortices to Multimodal semantic control: exploring refractory effects in semantic aphasia. J. Cognit. Neurosci..

[bib49] Gauthier L., Dehaut F., Joanette Y. (1989). The bells test: a quantitative and qualitative test for visual neglect. Int. J. Clin. Neuropsychol..

[bib50] Giora R. (1997). Understanding figurative and literal language: the graded salience hypothesis. Cognit. Linguist..

[bib51] Giora R., Zaidel E., Soroker N., Batori G., Kasher A. (2000). Differential effects of right- and left-hemisphere damage on understanding sarcasm and metaphor. Metaphor Symb..

[bib52] Gotts S.J., Plaut D.C. (2002). The impact of synaptic depression following brain damage: a connectionist account of “access/refractory” and “degraded-store” semantic impairments. Cognit. Affect. Behav. Neurosci..

[bib53] Hampshire A., Chamberlain S.R., Monti M.M., Duncan J., Owen A.M. (2010). The role of the right inferior frontal gyrus: inhibition and attentional control. NeuroImage.

[bib54] Harciarek M., Heilman K.M. (2009). The contribution of anterior and posterior regions of the right hemisphere to the recognition of emotional faces. J. Clin. Exp. Neuropsychol..

[bib55] Harvey D.Y., Schnur T.T. (2015). Distinct loci of lexical and semantic access deficits in aphasia: evidence from voxel-based lesion-symptom mapping and diffusion tensor imaging. Cortex.

[bib56] Hoffman P., Jefferies E., Ehsan S., Hopper S., Lambon Ralph M.A. (2009). Selective short-term memory deficits arise from impaired domain-general semantic control mechanisms. J. Exp. Psychology: Learn., Mem. Cogn..

[bib57] Hoffman P., Jefferies E., Ehsan S., Jones R.W., Lambon Ralph M.A. (2012). How does linguistic knowledge contribute to short-term memory? Contrasting effects of impaired semantic knowledge and executive control. Aphasiology.

[bib58] Hoffman P., Jefferies E., Lambon Ralph M.A. (2011). Explaining semantic short-term memory deficits: evidence for the critical role of semantic control. Neuropsychologia.

[bib59] Hoffman P., Jones R.W., Lambon Ralph M.A. (2012). The degraded concept representation system in semantic dementia: damage to pan-modal Hub, then visual spoke. Brain.

[bib60] Hutsler J., Galuske R.A.W. (2003). Hemispheric asymmetries in cerebral cortical networks. Trends Neurosci..

[bib61] Jefferies E. (2013). The neural basis of semantic cognition: converging evidence from neuropsychology, Neuroimaging and TMS. Cortex.

[bib62] Jefferies E., Baker S.S., Doran M., Lambon Ralph M.A. (2007). Refractory effects in stroke aphasia: a consequence of poor semantic control. Neuropsychologia.

[bib63] Jefferies E., Hoffman P., Jones R.W., Lambon Ralph M.A. (2008). The impact of semantic impairment on verbal short-term memory in stroke aphasia and semantic dementia: a comparative study. J. Mem. Lang..

[bib64] Jefferies E., Lambon Ralph M.A. (2006). Semantic impairment in stroke aphasia versus semantic dementia: a case-series comparison. Brain.

[bib65] Jefferies E., Patterson K., Jones R.W., Lambon Ralph M.A. (2009). Comprehension of concrete and abstract words in semantic dementia. Neuropsychology.

[bib66] Jefferies E., Patterson K., Lambon Ralph M.A. (2008). Deficits of knowledge versus executive control in semantic cognition: insights from cued naming. Neuropsychologia.

[bib67] Jung-Beeman M. (2005). Bilateral brain processes for comprehending natural language. Trends Cognit. Sci..

[bib68] Kacinik N.A., Chiarello C. (2007). Understanding metaphoric language: is the right hemisphere uniquely involved?. Brain Lang..

[bib69] Kemmerer D., Rudrauf D., Manzel K., Tranel D. (2012). Behavioral patterns and lesion sites associated with impaired processing of lexical and conceptual knowledge of actions. Cortex.

[bib70] Kempler, D., Van Lancker, D.,1985. The Formulaic and Novel Language Comprehension Test (FANL-C).

[bib71] Kiss G.R., Armstrong C., Milroy R., Piper J., Aitkin A.J., Bailey R.W., Hamilton-Smith N. (1973). An associative thesaurus of English and its computer analysis. The Computer And Literary Studies.

[bib72] Klepousniotou E., Baum S.R. (2005). Unilateral brain damage effects on processing homonymous and polysemous Words. Brain Lang..

[bib73] Kolb B., Taylor L., Lane R.D., Nadel L. (2000). Facial expression, emotion and hemispheric organization. Cognitive Neuroscience of Emotion.

[bib74] Krieger-Redwood K., Teige C., Davey J., Hymers M., Jefferies E. (2015). Conceptual control across modalities: graded specialisation for pictures and words in inferior frontal and posterior temporal cortex. Neuropsychologia.

[bib75] Kucharska-Pietura K., Phillips M.L., Gernand W., David A.S. (2003). Perception of emotions from faces and voices following unilateral brain damage. Neuropsychologia.

[bib76] Lambon Ralph M.A. (2014). Neurocognitive insights on conceptual knowledge and its breakdown. Philos. Trans. R. Soc. B: Biol. Sci..

[bib77] Lambon Ralph M.A., Cipolotti L., Manes F., Patterson K. (2010). Taking both Sides: do unilateral anterior temporal lobe lesions disrupt semantic memory?. Brain.

[bib78] Lambon Ralph M.A., Ehsan S., Baker G.A., Rogers T.T. (2012). Semantic memory is impaired in patients with unilateral anterior temporal lobe resection for temporal lobe epilepsy. Brain.

[bib79] Lambon Ralph M.A., McClelland J.L., Patterson K., Galton C.J., Hodges J.R. (2001). No right to speak? The relationship between object naming and semantic impairment: neuropsychological evidence and a computational model. J. Cognit. Neurosci..

[bib80] Langner O., Dotsch R., Bijstra G., Wigboldus D.H.J., Hawk S.T., van Knippenberg A. (2010). Presentation and validation of the Radboud faces database. Cogn. Emot..

[bib81] Laures-Gore J., Marshall R.S., Verner E. (2011). Performance of individuals with left hemisphere stroke and aphasia and individuals with right brain damage on forward and backward digit span tasks. Aphasiology.

[bib82] Lee S.S., Dapretto M. (2006). Metaphorical Vs. Literal word meanings: fMRI evidence against a selective role of the right hemisphere. NeuroImage.

[bib83] Lenartowicz A., Verbruggen F., Logan G.D., Poldrack R.A. (2011). Inhibition-related activation in the right inferior frontal gyrus in the absence of inhibitory cues. J. Cognit. Neurosci..

[bib84] Levy B.J., Wagner A.D. (2011). Cognitive control and right Ventrolateral prefrontal cortex: reflexive reorienting, motor inhibition, and action updating. Ann. N. Y. Acad. Sci..

[bib85] Liljestrom M., Tarkiainen A., Parviainen T., Kujala J., Numminen J., Hiltunen J., Laine M., Salmelin R. (2008). Perceiving and naming actions and objects. NeuroImage.

[bib86] Martin R.C., Shelton J., Yaffee L.S. (1994). Language processing and working memory: neuropsychological evidence for separate phonological and semantic capacities. J. Mem. Lang..

[bib87] Mashal N., Faust M. (2009). Conventionalisation of novel metaphors: a shift in hemispheric asymmetry. Laterality: Asymmetries Body, Brain Cogn..

[bib88] Maurer D., Le Grand R., Mondloch C.J. (2002). The many faces of Configural processing. Trends Cognit. Sci..

[bib89] McCarthy R.A., Warrington E.K. (2015). Past, present and prospects: reflections 40 years on from the selective impairment of semantic memory. Q. J. Exp. Psychol..

[bib90] Mion M., Patterson K., Acosta-Cabronero J., Pengas G., Izquierdo-Garcia D., Hong Y.T., Fryer T.D., Williams G.B., Hodges J.R., Nestor P.J. (2010). What the left and right anterior fusiform gyri tell us about semantic memory. Brain.

[bib91] Mostofsky S.H., Simmonds D.J. (2008). Response inhibition and response selection: two sides of the same coin. J. Cognit. Neurosci..

[bib92] Mummery C.J., Patterson K., Price C.J., Ashburner J., Frackowiak R.S.J., Hodges J.R. (2000). A voxel-based morphometry study of semantic dementia: relationship between temporal lobe atrophy and semantic memory. Ann. Neurol..

[bib93] Myers P.S., Perkins W.H. (1983). Right hemisphere communication disorders. Current Therapy in Communication Disorders.

[bib94] Nakamura K., Kawashima R., Ito K., Sugiura M., Kato T., Nakamura A., Hatano K., Nagumo S., Kubota K., Fukuda H., Kojima S. (1999). Activation of the right inferior frontal cortex during assessment of facial emotion. J. Neurophysiol..

[bib95] Nelson, D.L., McEvoy, C.L., Schreiber,T.A., 1998. The University of South Florida Word Association, Rhyme, and Word Fragment Norms. 2015 〈http://www.usf.edu/FreeAssociation/〉 10.3758/bf03195588 15641430

[bib96] Noonan K.A., Jefferies E., Corbett F., Lambon Ralph M.A. (2010). Elucidating the nature of deregulated semantic cognition in semantic aphasia: evidence for the roles of prefrontal and temporo-parietal cortices. J. Cognit. Neurosci..

[bib97] Noonan K.A., Jefferies E., Visser M.E.J., Lambon Ralph M.A. (2013). Going beyond inferior prefrontal involvement in semantic control: evidence for the additional contribution of parietal and posterior middle temporal cortex. J. Cognit. Neurosci..

[bib98] Patterson K., Nestor P.J., Rogers T.T. (2007). Where do you know what you know? The representation of semantic knowledge in the human brain. Nat. Rev. Neurosci..

[bib99] Phan T.G., Donnan G.A., Wright P.M., Reutens D.C. (2005). A digital map of middle cerebral artery infarcts associated with middle cerebral artery trunk and branch occlusion. Stroke.

[bib100] Phan T.G., Fong A.C., Donnan G.A., Reutens D.C. (2007). Digital map of posterior cerebral artery infarcts associated with posterior cerebral artery trunk and branch occlusion. Stroke.

[bib101] Pobric G., Mashal N., Faust M., Lavidor M. (2008). The role of the right cerebral hemisphere in processing novel metaphoric expressions: a Transcranial magnetic stimulation study. J. Cognit. Neurosci..

[bib102] Rapp A.M., Leube D.T., Erb M., Grodd W., Kircher T.T.J. (2007). Laterality in metaphor processing: lack of evidence from functional magnetic resonance imaging for the right hemisphere theory. Brain Lang..

[bib103] Raven J.C. (1962). Coloured Progressive Matrices Sets A, AB, B.

[bib104] Rehak A., Kaplan J.A., Gardner H. (1992). Sensitivity to conversational deviance in right hemisphere damaged patients. Brain Lang..

[bib105] Reitan R.M. (1958). Validity of the trail making test as an indicator of organic brain damage. Percept. Mot. Skills.

[bib106] Rice, G.E., Lambon Ralph, M.A., Hoffman, P., 2015. The roles of left versus right anterior temporal lobes in conceptual knowledge: an ALE meta-analysis of 97 functional neuroimaging studies. Cereb. Cortex. 10.1093/cercor/bhv024 PMC4816787 25771223

[bib107] Rinaldi M.C., Marangolo P., Baldassarri F. (2004). Metaphor comprehension in right brain-damaged patients with visuo-verbal and verbal material: a dissociation (re)considered. Cortex.

[bib108] Robertson I.H., Ward T., Ridgeway V., Nimmo-Smith I. (1994). The Test of Everyday Attention.

[bib109] Robinson G., Shallice T., Bozzali M., Cipolotti L. (2010). Conceptual proposition selection and the LIFG: neuropsychological evidence from a focal frontal group. Neuropsychologia.

[bib110] Robinson G., Shallice T., Bozzali M., Cipolotti L. (2012). The differing role of the frontal cortex in fluency tests. Brain.

[bib111] Robinson G.A., Cipolotti L., Walker D.G., Biggs V., Bozzali M., Shallice T. (2015). Verbal suppression and strategy use: a role for the right lateral prefrontal cortex?. Brain.

[bib112] Rodd J.M., Davis M.H., Johnsrude I.S. (2005). The neural mechanisms of speech comprehension: fMRI studies of semantic ambiguity. Cereb. Cortex.

[bib113] Rogers T.T., Patterson K., Jefferies E., Lambon Ralph M.A. (2015). Disorders of representation and control in semantic cognition: effects of familiarity, typicality, and specificity. Neuropsychologia.

[bib114] Schmidt G.L., DeBuse C.J., Seger C.A. (2007). Right hemisphere metaphor processing? Characterizing the Lateralization of semantic processes. Brain Lang..

[bib115] Schnur T.T., Lee E., Coslett H.B., Schwartz M.F., Thompson-Schill S.L. (2005). When lexical selection gets tough, the LIFG gets going: a lesion analysis study of interference during word production. Brain Lang..

[bib116] Schnur T.T., Schwartz M.F., Brecher A., Hodgson C. (2006). Semantic interference during blocked-cyclic naming: evidence from aphasia. J. Mem. Lang..

[bib117] Schnur T.T., Schwartz M.F., Kimberg D.Y., Hirshorn E., Coslett H.B., Thompson-Schill S.L. (2009). Localizing interference during naming: convergent Neuroimaging and neuropsychological evidence for the function of Broca's area. Proc. Natl. Acad. Sci..

[bib118] Schwartz M.F., Dell G.S., Martin N., Gahl S., Sobel P. (2006). A case-series test of the interactive two-step model of lexical access: evidence from picture naming. J. Mem. Lang..

[bib119] Silberman E.K., Weingartner H. (1986). Hemispheric Lateralization of function related to emotion. Brain Cogn..

[bib120] Snowden J.S., Thompson J.C., Neary D. (2004). Knowledge of famous faces and Names in semantic dementia. Brain.

[bib121] Snyder H.R., Feigenson K., Thompson-Schill S.L. (2007). Prefrontal cortical response to conflict during semantic and phonological tasks. J. Cognit. Neurosci..

[bib122] Soni M., Lambon Ralph M.A., Noonan K.A., Ehsan S., Hodgson C., Woollams A.M. (2009). “L” is for tiger: effects of phonological (mis)cueing on picture naming in semantic aphasia. J. Neurolinguist..

[bib123] Soni M., Lambon Ralph M.A., Woollams A.M. (2011). “W” is for bath: can associative errors be cued?. J. Neurolinguist..

[bib124] Thompson-Schill S.L., D’Esposito M., Aguirre G.K., Farah M.J. (1997). Role of left inferior prefrontal cortex in retrieval of semantic knowledge: a reevaluation. Proc. Natl. Acad. Sci. USA.

[bib125] Thompson-Schill S.L., Swick D., Farah M.J., D’Esposito M., Kan I.P., Knight R.T. (1998). Verb generation in patients with focal frontal lesions: a neuropsychological test of Neuroimaging findings. Proc. Natl. Acad. Sci. USA.

[bib126] Thompson H.E., Robson H., Lambon Ralph M.A., Jefferies E. (2015). Varieties of semantic'access’ deficit in Wernicke’s aphasia and semantic aphasia. Brain.

[bib127] Tompkins C.A., Blake M.T., Wambaugh J., Meigh K. (2011). A novel, implicit treatment for language comprehension processes in right hemisphere brain damage: phase 1 data. Aphasiology.

[bib128] Tompkins C.A., Fassbinder W., Scharp V.L., Meigh K.M. (2008). Activation and maintenance of peripheral semantic features of unambiguous words after right hemisphere brain damage in adults. Aphasiology.

[bib129] Visser, M.E.J., Embleton, K.V., Lambon Ralph, M.A., 2012. Evidence for a caudo-rostral gradient of information convergence in the temporal lobes: an fMRI study of verbal and non-verbal semantic processing. J. Cogn. Neurosci.. 10.1162/jocn_a_00244 22621260

[bib130] Visser M.E.J., Jefferies E., Lambon Ralph M.A. (2010). Semantic processing in the anterior temporal lobes: a meta-analysis of the functional neuroimaging literature. J. Cognit. Neurosci..

[bib131] Wagner A.D., Paré-Blagoev E.J., Clark J., Poldrack R.A. (2001). Recovering meaning: left prefrontal cortex guides controlled semantic retrieval. Neuron.

[bib132] Warrington E.K., Cipolotti L. (1996). Word comprehension-the distinction between refractory and storage impairments. Brain.

[bib133] Warrington E.K., Crutch S.J. (2004). A circumscribed refractory access disorder: a verbal semantic impairment sparing visual semantics. Cognit. Neuropsychol..

[bib134] Warrington, E.K., James, M., 1991. The Visual Object and Space Perception Battery. Bury St Edmunds: Thames Valley Test Company.

[bib135] Warrington E.K., McCarthy R.A. (1983). Category specific access dysphasia. Brain.

[bib136] Watson C.E., Cardillo E.R., Ianni G.R., Chatterjee A. (2013). Action concepts in the brain: an activation likelihood estimation meta-analysis. J. Cognit. Neurosci..

[bib137] Wechsler D. (1987). Wechsler.

[bib138] Winner E., Gardner H. (1977). Comprehension of metaphor in brain-damaged patients. Brain.

[bib139] Witteman J., van Ijzendoorn M.H., van de Velde D., van Heuven V.J.J.P., Schiller N.O. (2011). The nature of hemispheric specialization for linguistic and emotional prosodic perception: a meta-analysis of the lesion literature. Neuropsychologia.

[bib140] Yang F.G., Edens J., Simpson C., Krawczyk D.C. (2009). Differences in task demands influence the hemispheric Lateralization and neural correlates of metaphor. Brain Lang..

[bib141] Zaidel E., Kasher A., Soroker N., Batori G. (2002). Effects of right and left hemisphere damage on performance of the “Right hemisphere communication Battery”. Brain Lang..

