# Supplementary material for: The role of the right hemisphere in semantic control: A case-series comparison of right and left hemisphere stroke
Source: Neuropsychologia. 2016 May;85:44–61. doi: 10.1016/j.neuropsychologia.2016.02.030 (PMC4863527; doi:10.1016/j.neuropsychologia.2016.02.030)
Supplement: Supplementary material [file mmc1.docx]

Table S1: Statistical comparison of lesion damage

|  | RH vs. mild SA | RH vs. SA | Mild SA vs. SA |
| --- | --- | --- | --- |
| BA9 | P = 1 | P = .306 (SA > RH) | P = .237 (SA > mild SA) |
| BA46 | P = .755 | P = .395 (SA > RH) | P = .680 |
| BA47 | P = .755 | P = .142 (SA > RH) | P = .267 (SA > mild SA) |
| BA45 | P = .755 | P = .612 | P = .783 |
| BA44 | P = .268 (mild SA > RH) | P = .735 | P = .332 (mild SA > SA) |
| BA6 | P = .876 | P = .612 | P = .490 (mild SA > SA) |
| BA22 | P = .876 | P = .230 (RH > SA) | P = .298 (mild SA > SA) |
| BA21 | P = .639 | P = .933 | P = .535 |
| BA20 | P = 1 | P = .197 (SA > RH) | P = .142 (SA > mild SA) |
| BA36 | P = 1 | P = .672 | P = .630 |
| BA37 | P = .876 | P = .553 | P = .210 (SA > mild SA) |
| BA39 | P = .876 | P = .672 | P = .447 |
| BA40 | P = .755 | P = .866 | P = .731 |
| BA39 | P = 1 | P = .672 | P = .630 |
| BA19 | P = 1 | P = .197 (SA > RH) | P = .142 (SA > mild SA) |

Kruskal-Wallis tests were run to compare groups using information about how damaged the area of location is (0, 1, 2). P values with p > .5 show which group have proportionally more damage. Significance values displayed here are not corrected for multiple comparisons.
